# Economic evaluation, resource utilisation, and associated economic burden of myopia management: a systematic literature review

**DOI:** 10.7189/jogh.15.04322

**Published:** 2025-12-29

**Authors:** Thom WL de Milliano, Kun Shi-van Wielink, Frederic Ernst, Nagarjuna Mulkalapalli, Ravali Pagidigummula, Christina Lymperopoulou

**Affiliations:** 1 Santen SA, Geneva, Switzerland; 2 Santen Pharmaceutical SA, Amsterdam, Netherlands; 3 Santen Pharmaceutical GmbH, Munich, Germany; 4 IQVIA, Hyderabad, India; 5 IQVIA, Athens, Greece

## Abstract

**Background:**

Myopia progression through childhood to early adulthood can cause serious visual complications and impose a significant economic impact due to its high prevalence and associated management costs. This systematic literature review evaluates the economic impact, health care resource utilisation (HCRU), and cost-effectiveness of current myopia interventions.

**Methods:**

The systematic literature review was performed via the OVID SP® platform (software version 04.07.00, Mumbai, India) covering literature from 2009–2024, with no geographic restriction. Economic evaluation studies used the Markov model and the risk-of-bias was assessed using the Drummond and Jefferson checklist. Abstracts and full texts were screened independently by two reviewers; uncertainties and disagreements were resolved through reconciliation or arbitration by a third independent reviewer.

**Results:**

A total of 20 studies were included: 12 cost-effectiveness, four HCRU, and eight for health care costs across different age groups, with a predominance of studies from East Asia (China, Hong Kong, Singapore). Among the paediatric population, the most cost-effective interventions included digital strategies for early prevention and screening, low-dose atropine (0.05%) with an incremental cost-effectiveness ratio of 234 USD/Spherical Equivalent Refraction reduction, and non-pharmacological defocus incorporated multiple segment lenses (7074 USD/quality-adjusted life year (QALY) gained). In adults, ranibizumab was cost-effective (35 288 USD/QALY gained) for pathologic myopia, while small incision lenticule extraction surgery yielded long-term savings (15 USD/QALY gained). Economic burden was largely driven by vision correction procedures and specialist visits, with notable regional and socioeconomic disparities in Spain and China. In adults, HCRU (frequent treatment, monitoring, hospitalisation, and emergency visits) was higher for myopic choroidal neovascularisation compared to paediatric population, and direct costs exceeded indirect costs.

**Conclusion:**

s Myopia presents a substantial economic burden, highlighting the need for optimising cost-effective interventions to reduce severity, prevent long-term vision loss, and lessen the financial burden. However, limited data, geographical bias, methodological inconsistencies, and heterogeneity in outcomes emphasise the need for more standardised, comprehensive evaluations to ensure broader applicability.

Myopia, or short-sightedness, is the most common ocular disease globally [1]. It occurs when images are focused in front of the retina rather than directly on its surface, causing blurred vision of distant objects [1]. Risks for paediatric myopia include genetic predisposition, limited outdoor activity, prolonged near-work activities (reading or screen time), higher educational attainment, and urban living environments [2]. Myopia typically develops and progresses rapidly during childhood (ages 6–14), and stabilises between 15–20 years, although progression might continue for some individuals up to their early 20s [3–5]. Sometimes, continued progression leads to high myopia (HM), defined as a spherical equivalent of≤−6.0 dioptres (D) across most guidelines, including the American Optometric Association and the European Society of Ophthalmology, or an axial length of 26.5 mm or more in either eye [6–8]. High myopia is associated with visually impairing complications such as myopic macular degeneration, retinal degeneration and detachment, open-angle glaucoma, and early-onset cataracts [1,9,10], with some developing pathologic myopia (PM), a severe form characterised by progressive structural changes in the posterior segment of the eye [11]. Pathologic myopia can lead to myopic choroidal neovascularisation (mCNV), which affects approximately 5–11% of individuals with PM, causing irreversible central vision loss if untreated [12].

Myopia is a growing public health concern [13], with the global prevalence among children and adolescents estimated at 24.32% in 1990 and projected to reach 39.80% by 2050 [14]. As of 2023, the global estimated prevalence was 30.47%, with the highest in East Asia (35.22%), followed by Europe (22.60%), North America (21.17%), Oceania (7.93%), Africa (4.77%), Latin America, and the Caribbean (3.75%) [14]. In East Asia, the prevalence in China increased from 1.76% at age four to 84.6% at age 17 in 2013 [15]. Urban residency is associated with higher myopia rates [14], such as in Singapore, where 81.6% of young males are affected [16]. Notably, females (33.57%), adolescents (47.00%), and high school students (45.71%) show a high prevalence globally [14].

Current management strategies for childhood myopia primarily focus on optimising environmental influences, pharmacological treatments, and optical devices [13]. Optical devices such as orthokeratology (OrthoK), multifocal soft contact lenses (SCL), and peripheral defocus spectacles are commonly used to slow myopia progression in children [17]. Low-dose atropine has demonstrated efficacy and safety in slowing the progression of paediatric myopia [18,19]. For adults, treatment options include myopia correction and complication management through conventional glasses and contact lenses, laser-assisted in situ keratomileusis, photorefractive keratectomy, small incision lenticule extraction (SMILE), and implantable collamer lens [20]. Pharmacological treatments, such as antivascular endothelial growth factor agents, are used for managing complications such as mCNV in adults [21].

The global economic burden of myopia and its associated complications is substantial and rising [22], driven by an increasing prevalence and management costs [23]. In 2018, the global costs associated with myopia-related blindness, including direct health care expenses, productivity losses, and social security costs, exceeded 670 billion USD [24]. These costs are projected to reach 1.7 trillion USD by 2050 [24]. Productivity losses due to uncorrected myopia and severe vision impairment further amplify the overall economic impact [22].

Given the increasing prevalence and significant economic impact of myopia, evaluating the cost-effectiveness of interventions and the economic burden of current management is crucial. Recently, several innovative interventions for myopia have been introduced, with several others currently in development. While most of these interventions are accessible only in specific regions, assessing their cost-effectiveness is essential to optimise the use of available health care resources [21,25,26]. This systematic literature review (SLR) aims to assess published evidence on the economic evaluation of myopia treatment strategies, health care resource utilisation (HCRU), and the economic burden of preventing and managing myopia and its complications.

## METHODS

This SLR was conducted in accordance with the Cochrane Handbook, Centre for Reviews and Dissemination (University of York), Preferred Reporting Items for Systematic reviews and Meta-Analyses guidelines, and National Institute for Health and Care Excellence recommendations. While the review was not registered in prospective register of systematic reviews, at the time of planning, the scope and timeline of the review were aligned with internal quality assurance processes and stakeholder requirements, which did not mandate external registration.

### Eligibility criteria and search strategy

The eligibility criteria for study inclusion were defined according to the PICOS (population, interventions, comparisons, and outcomes, and study design) framework, publication year, and language limits, with no age or geographic restrictions. Economic evaluation studies comprised cost-effectiveness, -utility, -benefit, -consequence, and -minimisation analyses. Conference abstracts published before 2022 and studies not reporting relevant outcomes, animal studies, case reports, editorials, and comments were excluded (Table S1 in the **Online Supplementary Document**).

The search was conducted using the OVID SP® platform, with access to multiple databases, including the Embase®, MEDLINE®, CENTRAL®, and EconLit® databases, and covered publication years from 2009–2024. Additionally, hand searches of Health Technology Assessment websites, conference proceedings, and trial registries were performed to identify unpublished or ongoing studies. The search strategies combined free-text words and indexing terms related to myopia and study designs, using Boolean operators. Population search terms for myopia were combined with interventions (*e.g.* atropine, OrthoK, contact lenses), comparators, outcomes, and study designs, applying relevant limits.

### Study screening and selection

The screening process involved a two-step approach: abstract review, followed by full-text review. Abstracts were independently reviewed by two reviewers, based on the PICOS criteria, and 10% of the abstracts were quality checked by a third independent reviewer. At the full-text review stage, uncertainties and disagreements were resolved either through ‘reconciliation’ or ‘arbitration’ by a third independent reviewer, where the ‘majority view’ determined inclusion or exclusion for the SLR. Extraction of data on the outcomes of interest from full-text studies was performed using a standardised data extraction template in Microsoft Excel®. The study selection process was documented using a PRISMA flow diagram. Citations identified were managed using EndNote® software (version 20.0 Bld 14672, Clarivate Analytics, Philadelphia, Pennsylvania, USA). Duplicate records were removed before exporting the remaining citations to Microsoft Excel® for further screening and data extraction. Data extracted included study characteristics (country, design, intervention, comparator, currency, cost-year, *etc*.) and outcomes (QALYs, life-years (Lys), disability-adjusted life-years (DALYs), total cost, incremental cost-effectiveness ratio (ICER), incremental cost-utility ratio (ICUR), HCRU, *etc*.).

### Data values inflation and conversion

To enhance the comparability of the data, costs reported for any year prior to 2023 were inflated to 2024 values using country-specific inflation rates sourced from International Monetary Fund [27], and converted to USD, where applicable, using the 2024 currency exchange rates sourced from the World Bank [28]. Whenever the year of cost data was not reported, a year prior to the publication year was used as a proxy. It is to be noted that values reported in the article have been inflated and converted using this approach, while original values are present in the **Online Supplementary Document**, along with currency exchange rates and inflation rates for different countries (Table S2–3 in the **Online Supplementary Document**).

### Risk-of-bias assessment

The methodological quality of the included studies was assessed using the Drummond and Jefferson (1996) checklist for economic evaluations in line with the National Institute for Health and Care Excellence (NICE) guide to the methods of technology appraisal [29,30], consisting of 35 questions covering study design, data collection, and analysis. Each question was rated ‘yes,’ ‘no,’ ‘not clear,’ or ‘not applicable,’ generating an overall quality score for each study. Studies were categorised as high-, moderate-, or low-quality based on their scores. High-quality studies met nearly all criteria, moderate-quality studies met most criteria but had some shortcomings, and low-quality studies failed to meet several criteria, such as inappropriate study design, lack of randomisation, or inadequate statistical analysis. The quality assessment of each study was carried out using the Drummond checklist for economic evaluation studies (Table S4 in the **Online Supplementary Document**).

## RESULTS

### Overall search results

**Figure 1:**
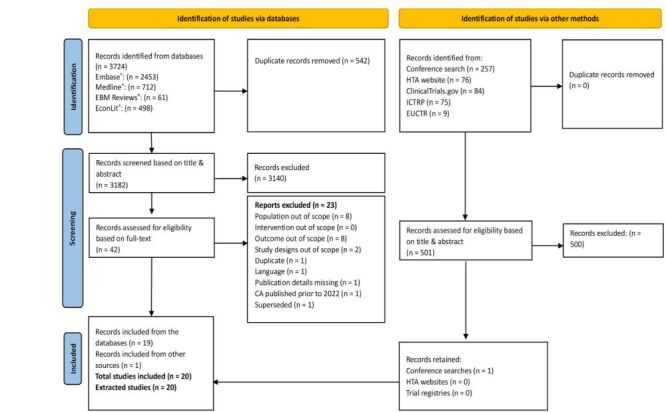
PRISMA flow diagram of screening and included studies. CA – conference abstracts, EBM – evidence-based medicine, EUCTR – European Union clinical trials register, HTA – health technology assessment, ICTRP – international clinical trials registry platform, PRISMA – Preferred Reporting Items for Systematic reviews and Meta-Analyses.

A total of 3724 references were identified from electronic database searches conducted on 22 April 2024 (MEDLINE®: 712; Embase®: 2453; EBM reviews®: 61; EconLit: 498). After removing duplicates, titles and abstracts of 3182 references were screened, resulting in 42 references for full-text screening. Finally, 19 relevant publications were identified, along with one additional record from conference screenings (2022–2024). The study disposition is depicted using a PRISMA flow diagram (**Figure 1**).

Out of the total 20 included publications, six studies were on paediatric and adolescent patients (≤18 years), 12 studies on adults (>18 years), and two studies involved all age groups. The included studies have been categorised based on population and reported outcomes, and the distribution has been mapped across geographies (Figure S1–2 in the **Online Supplementary Document**).

### Paediatric and adolescent populations (≤18 years)

#### Economic evaluation

Among the five studies reporting economic modelling outcomes in the ≤18 years age group [26,31–34], four were cost-effectiveness analyses (CEAs) [26,31–33], while one included both CEA and cost-utility analysis (CUA) [34]. All studies used the Markov model for economic evaluation, similar to the approach observed in previous myopia-related studies [20,26,31–34]. These analyses were performed in mainland China, Hong Kong, and New Zealand (Figure S2 in the **Online Supplementary Document**). The characteristics (population, region, intervention/comparator, modelling approach, type of economic analysis, time horizon, sensitivity analysis, and outcomes) of economic evaluation studies have been summarised (Table S5 in the **Online Supplementary Document**).

The quality assessment using the Drummond checklist of the five included studies showed that two were of high quality [32,34], while three were of medium quality (Table S4 in the **Online Supplementary Document**) [26,31,33].

A Chinese study reported outcomes of myopia screening and prevention in schoolchildren. Prevention and control strategies that implemented myopia awareness through digital health education channels (*e.g*. science articles) and mobile technology (*e.g*. short messages) were found to be more cost-effective than traditional methods such as health education activities and outdoor engagement initiatives [34]. The CUA showed that in rural settings, both traditional (ICUR = 7306 USD/QALY gained; inflation adjusted) and digital control strategies (ICUR = 13 072 USD/QALY gained; inflation adjusted) were cost-effective at the rural threshold of 30 501 USD/QALY gained. In urban settings, the digital strategy was cost-effective, with an ICUR = 12 334 USD/QALY gained (inflation adjusted), which is below the urban threshold of USD = 41 568/QALY gained. However, in the CEA, the ICERs for the digital strategy (43 221 USD/DALY averted in rural settings, 48 250 USD/DALY averted in urban settings; inflation adjusted) slightly exceeded the respective cost-effectiveness willingness-to-pay (WTP) thresholds (rural: 30 501 USD/QALY gained; urban: 41 568 USD/QALY gained) (**Table 1**) [34].

**Table 1:** Economic evaluations and health care costs findings for paediatric and adolescent populations

Study details	Intervention *vs*. comparator	*†Cost-effectiveness ICER (USD, 2024)	†WTP threshold (USD); cost-effectiveness
Li 2023; Cost-year NR; Mainland China [34]	Rural setting		
	DCMP *vs*. TMPC	ICUR: 13 072/QALY gained	30 501/QALY gained; cost-effective
		ICER: 43 221/DALY averted	30 501/DALY averted; not cost-effective
	TMPC *vs*. SMSP	ICUR: 7306/QALY gained	30 501/QALY gained; cost-effective
		ICER: 14 592/DALY averted	30 501/DALY averted; cost-effective
	Urban setting		
	DCMP *vs*. TMPC	ICUR: 12 334/QALY gained	41 568/QALY gained; cost-effective
		ICER: 48 250/DALY averted	41 568/DALY averted; not cost-effective
	TMPC *vs*. SMSP	ICUR: 17 704/QALY gained	41 568/QALY gained; cost-effective
		ICER: 21 213/DALY averted	41 568/DALY averted; cost-effective
Agyekum 2023; Cost-year 2022; Hong Kong [26]‡	Atropine 0.05% *vs*. SVLs	234/SER reduction	2506/SER reduction to 10 024/SER reduction; cost-effective
	Atropine 0.01% *vs*. SVLs	292/SER reduction	2506/SER reduction to 10 024/SER reduction; cost-effective
Agyekum 2023; Cost-year NR; Hong Kong [31]	Atropine 0.05% *vs*. NR	119/SER reduction§	WTP: NR
	Atropine 0.025% *vs*. NR	186/SER reduction§	WTP: NR
	Atropine 0.01% *vs*. NR	247/SER reduction§	WTP: NR
Lian 2023; Cost-year NR; Hong Kong [33]	DIMS *vs*. no myopia control	7074/QALY gained	48 161/QALY gained; cost-effective
Hong 2022; Cost-year 2021; New Zealand [32]	Photorefraction screening + atropine 0.01% *vs*. usual care	1006/QALY gained	41 024/QALY gained; cost-effective
Health care costs (direct)			
Study details	Cost description	Cost (USD)	
Lim 2009; Cost-year NR; Singapore [35]	Mean annual cost per person	238	

DCMP – digital comprehensive myopia prevention, DIMS – defocus incorporated multiple segments spectacle lens, ICER – incremental cost-effectiveness ratio, ICUR – Incremental Cost-Utility Ratio, NR – not reported, QALY – quality-adjusted life year, SER – spherical equivalent refraction, SMSP – school-based myopia screening, SVL – single vision lens, TMPC – traditional multi-prevention and control, WTP – Willingness-to-pay

*None of the studies reported data on the life-years gained or incremental life-years.

†The values reported in the table have been inflated and converted to USD based on the approach described in the Methods section.

‡Unit of measure is dioptres for spherical equivalent refraction.

§These ICER values are not converted or inflation adjusted due to lack of data on costs and SER reduction in the study.

Among pharmacological interventions, low-dose atropine consistently proved to be a cost-effective option for controlling myopia progression in children across three studies [26,31,32]. In Hong Kong, 0.05% atropine was most cost-effective with an ICER = 119 USD/Spherical Equivalent Refraction (SER) reduction (WTP threshold not reported) followed by ICERs of 186 USD/SER reduction and 247 USD/SER reduction for 0.025 and 0.01%, respectively (inflation unadjusted) [31]. Another Hong Kong study reported cost-effectiveness of 0.05% atropine at an ICER of 234 USD/SER reduction (inflation adjusted and WTP threshold ranged from 2506–10 024 USD/SER reduction) [26], while in New Zealand, photorefraction screening at age 11, followed by atropine 0.01%, was cost-effective with ICER = 1006 USD/QALY gained (inflation adjusted) at the WTP threshold of 41 024 USD/QALY gained (**Table 1**) [32].

Non-pharmacological methods, such as outdoor activity, and other interventions, such as red-light therapy, OrthoK, highly aspherical lenslets, and defocus incorporated multiple segments spectacle lens (DIMS), were cost-effective in Hong Kong [26,31,33]. The key findings from economic evaluation studies, with further details are summarised (**Table 1**; Table S6 in the **Online Supplementary Document**).

#### Health care costs

In the single study that reported health care cost outcomes, the mean annual direct health care cost for childhood myopia in Singapore was estimated at 238 USD per patient (inflation adjusted) [35]. Among the cost components, contact lenses incurred higher expenses compared to spectacles and optometrist visits. Notably, higher annual costs were observed among patients from families with higher total household income and those whose fathers had attained higher educational levels (*P* = 0.03 and *P* = 0.001, respectively). Additionally, children from higher-income households demonstrated a higher frequency of replacing spectacles (*P* = 0.02) and a shorter interval since the last change of spectacles (*P* = 0.03) [35].

A summary of health care cost findings for paediatric and adolescent populations and further details have been provided (**Table 1**; Table S6 in the **Online Supplementary Document**). No HCRU outcomes were reported in any of the studies identified in this review for paediatric and adolescent populations.

### Adult population (>18 years)

#### Economic evaluation

Among the seven studies that reported economic modelling outcomes in the adult population [21,36–41], five studies were CEA [21,36,39–41], one included both CEA and CUA [37], and one was a budget-impact analysis (BIA) [38]. The predominant modelling approach used was the Markov model [21,37,39–41], followed by the decision-tree model [36]. The BIA study adopted an open cohort model [38]. These studies were conducted across mainland China, Spain, the UK, and specifically in England and Wales (Figure S2 in the **Online Supplementary Document**). Detailed characteristics of these economic evaluations are presented (Table S5 in the **Online Supplementary Document**). Quality assessment revealed that one study was of high quality [37], two were of medium quality [21,36], and four were of poor quality [38–41]. The studies rated as low-quality were primarily conference abstracts with limited methodological details (Table S4 in the **Online Supplementary Document**) [38–41].

Given the increased risk of eye complications with advanced age, a Chinese study evaluated screening and prevention strategies in adults aged ≥50 years. The findings indicated that combined population-based screening for multiple eye diseases was more cost-effective than no screening [37]. Among the evaluated strategies, artificial intelligence-enabled telemedicine screening emerged as most cost-effective in urban settings with ICER of 3112 USD/y of blindness avoided (inflation adjusted), which was lower than the WTP threshold of 37 259 USD/QALY gained. Additionally, in rural settings, this approach dominated no screening strategy at the WTP threshold of 25 751 USD/QALY gained [37].

For myopia management, SMILE was identified as the most cost-effective (ICER = 15 USD/QALY gained; inflation adjusted) refractive correction surgery option at the WTP threshold of 25 128–28 555 USD/QALY gained in a Spanish population, compared to FS- laser-assisted in situ keratomileusis and photorefractive keratectomy (**Table 2**) [36].

**Table 2:** Economic evaluations, costs, and HCRU findings for adult population

Study details	Study population	Intervention *vs*. comparator		*†ICER (USD, 2024)	†WTP (USD); cost-effectiveness
Zhang 2019; Cost-year NR; Mainland China [41]	mCNV	Conbercept *vs*. Ranibizumab (Scenario 1‡)		NR	Three times GDP per capita; cost-effective
		Conbercept *vs*. Ranibizumab (Scenario 2‡)		NR	Three times GDP per capita; dominant
Liu 2019; Cost-year NR; Mainland China [40]	CNV secondary to PM	Ranibizumab *vs*. vPDT		35,288/QALY gained	37 259/QALY gained; cost-effective
Cui 2021; Cost-year NR; Mainland China [21]	PM	Ranibizumab *vs*. Conbercept		Dominant (cost/QALY gained)	30 819/QALY gained; dominant
Liu 2023; Cost-year 2021; Mainland China [37]	Multiple eye diseases (AMD, glaucoma, diabetic retinopathy, cataracts, and PM)	Rural setting			
		AI telemedicine screening *vs*. no screening		Dominant (cost/blindness year avoided)	25 751/QALY gained; dominant
		Urban setting			
		AI telemedicine screening *vs*. no screening		3112/blindness year avoided	37 259/QALY gained; cost-effective
Leteneux 2013; Cost-year NR; UK [39]	CNV secondary to PM	Ranibizumab *vs*. vPDT		NR	31 593/QALY gained; dominant
Balgos 2022; Cost-year 2020; Spain [36]	Adult myopia	SMILE *vs*. NR		15/QALY gained	25 128–28 555/QALY gained cost-effective
		PRK *vs*. NR		19/QALY gained	
		FS-LASIK *vs*. NR		15/QALY gained	
Health care costs					
Study details	Population	Sub-population	Cost unit	Cost category	†Cost (USD, 2024)
Direct costs					
¶Yang 2017; Cost-year 2011; Taiwan [42]	mCNV	NA	Mean total cost per visit	1st-year treatment visit§	vPDT: 23.34, IVI: 82.28
				2nd-year treatment visit§	vPDT: 17.40, IVI: 59.04
				1st-year monitoring visit¶	vPDT: 258.47, IVI: 270.81
				2nd-year monitoring visit¶	vPDT: 195.43, IVI: 215.27
Zheng 2013; Cost-year 2011; Singapore [43]	Myopia	NA	Mean annual PPPY cost	Total costs	898.11
				Optometry visits and use of spectacles & contact lenses	586.05
Ruiz-Moreno 2016; Cost-year 2014; Spain [44]	mCNV or myopia without mCNV	mCNV	Annual mean per patient cost	Medical costs	mCNV: 2629.38 Without mCNV: 472.07
				Non-medical costs	mCNV: 339.62 Without mCNV: 24.64
				Total cost	mCNV: 2968.99 Without mCNV: 496.71
			Annual mean per patient cost	Medical transportation (ambulances)	mCNV: 4.90 Without mCNV: 0.00
		NA	Total cost	Total average of all regions	2327.51
				Centre-Interior region	3174.17
				South region	2083.13
				East-Levante region	1952.79
				North-Cantabrian region	1532.11
Zaour 2014; Cost-year NR; Canada [45]	CNV secondary to PM, or mCNV	NA	Mean per patient cost	Mild vision loss	2858.88
				Moderate vision loss	3467.50
				Severe vision loss	3916.00
				Total average	3311.24
Indirect costs					
Ruiz-Moreno 2016; Cost-year 2014; Spain [44]	mCNV or myopia without mCNV	mCNV	Annual mean per patient cost	Indirect cost	5434.52
		Myopia without mCNV			5454.52
Naidoo 2017; Cost-year NR; Global [46]	Myopia and MMD	Uncorrected myopia	Global cost, billion per year	Productivity loss cost (including informal carer cost)	579.00
		MMD			4.46
HCRU					
Study details	Population	Description	Resource category		^Value
Yang 2017ǁ; Taiwan [42]	mCNV	Mean	1st-year treatment visit§		vPDT: 1.22 IVI: 1.85
			2nd-year treatment visit§		vPDT: 1.06 IVI: 1.73
			1st-year monitoring visit¶		vPDT: 5.65 IVI: 5.11
			2nd-year monitoring visit¶		vPDT: 4.13 IVI: 3.78
Ruiz-Moreno 2016; Spain [44]	mCNV or myopia without mCNV	Mean number of visits	Ophthalmologist visit		mCNV: 7.60 Without mCNV: 8.80
			Retina specialists visit		mCNV: 10.70 Without mCNV: 9.00
			Emergency department visit		mCNV: 1.50 Without mCNV: 1.70
		HCRU, n (%)	Hospitalisation		mCNV: 0 (0.00) Without mCNV: 2 (4.20)
			Ophthalmologist visit		mCNV: 95 (67.20) Without mCNV: 36 (75)
			Retina specialists visit		mCNV: 125 (91.20) Without mCNV: 37 (77.10)
			Emergency department visit		mCNV: 57 (41.70) Without mCNV: 12 (25)
		Mean	Absenteeism (days; within the past 12 mo)		mCNV: 21.80 Without mCNV: 0.00
			Presenteeism (days; within the past six months)		mCNV: 6.70 Without mCNV: 1.20
Zaour 2014; Canada [45]	CNV secondary to PM, or mCNV	Mean; n (%)	Retina specialist consultation, per year		6; 88 (90)
		n (%)	Emergency department visit		11 (11.20)

AI – artificial intelligence, AMD – age-related macular degeneration, CNV – choroidal neovascularisation, FS-LASIK – femtosecond laser-assisted in situ keratomileusis, GDP – gross domestic product, HCRU – health care resource utilisation, ICER – incremental cost-effectiveness ratio, IVI – intravitreal antivascular endothelial growth factor injections, mCNV – myopic choroidal neovascularisation, MMD – myopic macular degeneration, NA – not applicable, NR – not reported, PM – pathologic myopia, PPPY – per patient per year, PRK – photorefractive keratectomy, QALYs – quality-adjusted life-years, SMILE – small incision lenticule extraction, TWD – New Taiwan dollar, vPDT – verteporfin photodynamic therapy, WTP – Willingness-to-pay

*None of the studies reported data on the life-years gained or incremental life-years.

†The values reported in the table have been inflated and converted to USD based on the approach described in the Methods section.

‡Scenario 1: The number of injections in two arms were from the clinical trials; Scenario 2: The number of injections in two arms were from real-world studies.

§Administration (non-drug) cost per visit, including examination, diagnosis, and concomitant drug.

¶Cost per monitoring visit including diagnosis, examination, and others.

ǁYang 2017: 1 USD = 30.3 TWD.

Among the five studies (one BIA and four cost-effectiveness model) focusing on the management of adult myopia complications [21,38–41], three compared ranibizumab with verteporfin photodynamic therapy (vPDT) in patients with mCNV [38–40], while two compared ranibizumab with conbercept [21,41]. The BIA conducted in Wales and England showed that although initial annual treatment costs for visual impairment due to mCNV were higher with ranibizumab compared to vPDT, substantial cost savings were observed at Year 3 (7093 USD), Year 4 (223 000 USD), and Year 5 (426 373 USD), amounting to a cumulative five-year savings of 416 503 USD (all inflation adjusted) [38]. Other studies in UK and China showed similar findings (**Table 2**) [39,40].

In China, conbercept favoured over ranibizumab for treating PM due to its less cost and more effectiveness. Compared with ranibizumab, the incremental effectiveness of conbercept was 0.029 QALYs [21,41]. In a separate study, conbercept was deemed cost-effective under the threshold of three times gross domestic product per capita [41]. Clinical trial data showed inflation adjusted total costs and QALYs of 27 860 USD and 9.86, respectively, for conbercept, compared to 27 203 USD and 9.83, respectively, for ranibizumab [41]. In real-world studies, QALYs remained constant, but conbercept incurred lower costs than ranibizumab (182 474 *vs*. 183 516 USD, respectively; inflation adjusted), thereby making it a preferred choice. Probabilistic sensitivity analysis indicated a 53.00% chance of conbercept being cost-effective in clinical trials and a 66.30% chance in real-world studies [41], thereby suggesting conbercept to be marginally cost-effective (**Table 2**).

Key economic evaluation findings for adults and further details are provided (**Table 2**; Table S7 in the **Online Supplementary Document**).

#### Health care costs

Health care costs in the adult population were analysed in five studies [42–46]. Overall, direct costs associated with myopia management were noted to be high in case of myopia-related complications and due to spectacles and lens usage.

**Figure 2:**
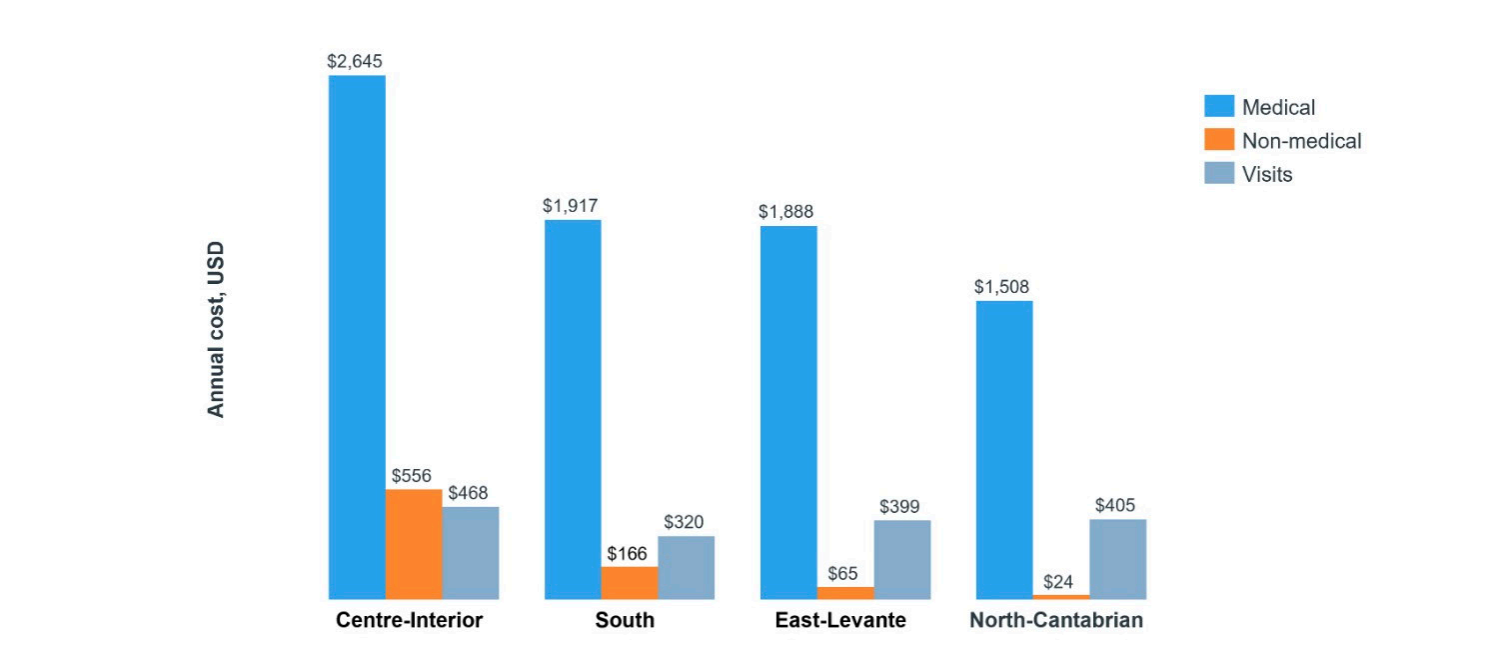
Annual cost of medical, non-medical, and visits in various regions of Spain.

In the Spanish study, the financial burden for mCNV was found to be higher than for myopia patients without mCNV. Annual medical costs for mCNV patients were 2 629.38 USD (inflation adjusted) compared to 472.07 USD (inflation adjusted) for myopia patients without mCNV. The total annual costs were 2 968.99 USD (inflation adjusted) for mCNV patients *vs*. 496.71 USD (inflation adjusted) for myopia patients without mCNV, including costs for emergency department visits (ER) (41.70 *vs*. 25.00%; *P* = 0.06), retinal specialists (91.20 *vs*. 77.10%; *P* = 0.01), medical consultations, and screening/follow-up tests (**Table 2**) [44]. Notable regional disparities were observed in health care expenditure across four Spanish regions, with the Centre-Interior area reporting the highest overall costs and per visit costs, while the North-Cantabrian region incurred the lowest costs [44]. These regional variations and the key costs, with details are provided (**Figure 2**, **Table 2**; Table S7 in the **Online Supplementary Document**).

In the study conducted in Singapore, the mean annual cost of treating myopia per adult was 898.11 USD (inflation adjusted), with spectacles, contact lenses, and optometry services being the major cost drivers, contributing to approximately 65% of the total expenditure. The total cost of myopia for the entire country was 843 million USD per year (inflation adjusted) [43].

In Taiwan, administrative costs for intravitreal antivascular endothelial growth factor injections (IVI) was higher (mean = 82.28 USD, inflation adjusted) per treatment visit along with high monitoring costs, while vPDT incurred higher overall treatment visit costs (Figure S3, Panels A–B in the **Online Supplementary Document**) [42].

A study conducted in Canada involving patients with mCNV complications secondary to PM reported the annual average mCNV-related cost per patient at 2859 USD (inflation adjusted). The cost of care varied with the severity of vision loss, ranging from 2859–3916 USD (inflation adjusted) for mild-to-severe vision loss [45].

In 2015, indirect costs reflected by the global economic productivity loss due to uncorrected myopia was 579 USD billion per year (inflation adjusted), while myopic macular degeneration contributed to losses of 4.46 billion USD per year (inflation adjusted) (**Table 2**) [46].

In the Spanish study, mean annual indirect costs per patient were comparable between patients with HM with or without mCNV (5434.52 *vs*. 5454.52 USD, respectively; inflation adjusted). However, a higher proportion of patients with mCNV reported a moderate-to-severe impact on work life (27.7 *vs*. 10.4%), and mCNV significantly impaired activity (odds ratio (OR) = 3.47; 95% confidence interval (CI) = 10.101–1.195). While this implies a greater burden of productivity loss in patients with mCNV, the reported indirect costs were comparable to patients without mCNV (**Table 2**) [44].

Key health care cost findings for adults and further details are summarised (**Table 2**; Table S7 in the **Online Supplementary Document**).

#### HCRU

Only three studies reported HCRU outcomes in adults [42,44,45]. Resource utilisation patterns in patients with PM and mCNV varied across studies and included parameters such as treatment visits, monitoring visits, specialist consultations, hospitalisations, and productivity loss (**Table 2**; Table S7 in the **Online Supplementary Document**).

A Spanish study reported that patients with mCNV had a significantly higher mean number of visits to retinal specialists (10.70 *vs*. 9.00 visits, *P* < 0.001) and a greater proportion of such visits (91.20 *vs*. 77.1%, *P* = 2) compared with those without mCNV. For ER, average visits were 1.50 for patients with mCNV *vs*. 1.70 for those without, while the percentage of ER visits was 41.60 *vs*. 25.00%. Notably, no hospitalisations were reported among patients with mCNV, whereas 4.20% of patients without mCNV were hospitalised for cataract or retinal detachment surgery. Additionally, mCNV was associated with an average of 21.80 missed workdays annually and a mean productivity loss of 6.70 days over the previous six months, compared to no missed workdays and a productivity loss of 1.20 days in patients without mCNV (**Table 2**) [44].

In Taiwan, patients with mCNV receiving IVI had more frequent treatment visits with average annual visits of 1.85 in the first year and 1.73 in the second year, while vPDT was associated with a higher number of monitoring visits (5.65) initially; both therapies showed a decline in visit frequency over time [42].

In Canada, most mCNV patients (90%) consulted retinal specialists (average 6.0 visits/y), with a minority requiring ER visits (11.2%) or hospitalisation (2.0%) [45].

### All age groups

No economic evaluation outcomes were reported in studies involving all age groups, and health care costs were analysed in only two studies [47,48].

#### Direct health care costs

Regional disparities in medical service costs were observed among patients aged 5–50 years across different socioeconomic regions in China. Patients in Yunnan, a lower socioeconomic region, incurred higher annual costs for hospital-based medical services including medical cost, traffic cost, cost of working time loss, and accompanying cost compared to those in Anhui (middle socioeconomic region) and Shanghai (higher socioeconomic region). The annual mean medical costs were highest in Yunnan at 25.75 USD, followed by Shanghai at 23.42 USD and Anhui at 16.57 USD (all inflation adjusted). Higher traffic costs incurred by patients in Yunnan possibly reflect differences in transportation infrastructure and accessibility in these regions (**Table 3**) [47].

**Table 3:** Health care costs and HCRU findings for all age groups

Health care costs					
**Study details**	**Population**	**Subgroups**		**Description**	*Converted cost (USD, 2024)
**Direct costs**					
Ma 2022; Cost-year 2016; Mainland China [47]	Myopia 5–50 y	Patients residing in middle social economic area (Anhui)			
		Annual mean per patient cost		Cost of replacement of spectacles and contact lenses	Spectacles: 23.51; OrthoK and RGPCL = 569.08; SCL = 143.80
		Annual direct mean cost		Medical health service in hospital	Medical cost = 16.57; Traffic cost = 5.62; Myopic laser surgery = 1391.54
				Treatment outside hospital	36.04
				Prevention of myopia	64.23
		Patients residing in upper social economic area (Shanghai)			
		Annual mean per patient cost		Cost of replacement of spectacles and contact lenses	Spectacles = 47.55; OrthoK and RGPCL = 633.72; SCL = 263.24
		Annual direct mean cost		Medical health service in hospital	Medical cost = 23.42; Traffic cost = 3.35; Myopic laser surgery = 1608.98
				Treatment outside hospital	53.87
				Prevention of myopia	85.96
		Patients residing in lower social economic area (Yunnan)			
		Annual mean per patient cost		Cost of replacement of spectacles and contact lenses	Spectacles = 45.84; OrthoK and RGPCL = 606.22; SCL = 191.83
		Annual direct mean cost		Medical health service in hospital	Medical cost = 25.75; Traffic cost = 9.00; Myopic laser surgery = 2014.26
				Treatment outside hospital	58.29
				Prevention of myopia	58.53
Fricke 2022; Cost-year 2020; Australia & China [48]	Myopia all age groups	Mean costs		Australian myopia patients	TMM = 9134.10
					Anti-myopia spectacles = 8941.27
					Low-dose atropine = NR
				Chinese myopia patients	TMM = 9832.94
					Anti-myopia spectacles = NR
					Low-dose atropine = 5469.16
**Indirect costs**					
Ma 2022; Cost-year 2016; Mainland China [47]	Myopia 5–50 y	Mean cost of loss of productivity, in billions		Mild to moderate visual impairment	6.74
				Severe visual impairment to blindness	9.41
		Patients residing in middle social economic area (Anhui)			
		Annual direct mean cost		Medical health service in hospital	Cost of working time loss = 5.75; Cost of accompanying = 39.82; Cost of working time loss for myopic laser surgery = 235.99
				Treatment outside hospital	Cost of working time loss = 3.98; Cost of accompanying = 18.14
		Patients residing in upper social economic area (Shanghai)			
		Annual direct mean cost		Medical health service in hospital	Cost of working time loss = 10.74; Cost of accompanying = 73.22; Cost of working time loss for myopic laser surgery = 743.18
				Treatment outside hospital	Cost of working time loss = 10.76; Cost of accompanying = 42.83
		Patients residing in lower social economic area (Yunnan)			
		Annual direct mean cost		Medical health service in hospital	Cost of working time loss = 13.48; Cost of accompanying = 77.53; Cost of working time loss for myopic laser surgery = 492.39
				Treatment outside hospital	Cost of working time loss = 8.41; Cost of accompanying = 389.68
**HCRU**					
Reference	Population	Subgroups	Description		^Value
Ma 2022; Mainland China [47]	Myopia 5–50 y	Patients residing in middle social economic area (Anhui)			
		Overall (%)	Hospital visit		20.60
			Medical health service utilisation (aged 5–9)		64.20
			Medical health service utilisation (aged 10–14)		44.60
			Medical health service utilisation (aged 15–19)		23.80
		Mean	Frequency of replacement, years		Spectacles = 2.10; OrthoK and RGPCL = 1.20; SCL = NR
		%	Medical health service utilisation (aged 5–9)		Spectacles = 59.35; OrthoK and RGPCL = 2.49; SCL = NR
			Medical health service utilisation (aged 10–14)		Spectacles = 82.12; OrthoK and RGPCL = 3.11; SCL = 0.62
			Medical health service utilisation (aged 15–19)		Spectacles = 94.58; OrthoK and RGPCL = 1.02; SCL = 5.15
		Patients residing in upper social economic area (Shanghai)			
		Overall (%)	Hospital visit		16.80
			Medical health service utilisation (aged 5–9)		81.50
			Medical health service utilisation (aged 10–14)		55.40
			Medical health service utilisation (aged 15–19)		16.50
		Mean	Frequency of replacement, years		Spectacles = 2.40; OrthoK and RGPCL = 1.80; SCL = NR
		%	Medical health service utilisation (aged 5–9)		Spectacles = 62.34; OrthoK and RGPCL = 3.89; SCL = NR
			Medical health service utilisation (aged 10–14)		Spectacles = 79.95; OrthoK and RGPCL = 9.10; SCL = 1.14
			Medical health service utilisation (aged 15–19)		Spectacles = 92.96; OrthoK and RGPCL = 3.79; SCL = 8.64
		Patients residing in lower social economic area (Yunnan)			
		Overall (%)	Hospital visit		28.80
			Medical health service utilisation (aged 5–9)		73.30
			Medical health service utilisation (aged 10–14)		66.70
			Medical health service utilisation (aged 15–19)		37.40
		Mean	Frequency of replacement, years		Spectacles = 2.30; OrthoK and RGPCL = 1.60; SCL = NR
		%	Medical health service utilisation (aged 5–9)		Spectacles = 40.11; OrthoK and RGPCL = NR; SCL = NR
			Medical health service utilisation (aged 10–14)		Spectacles = 81.31; OrthoK and RGPCL = 5.50; SCL = 1.24
			Medical health service utilisation (aged 15–19)		Spectacles = 92.96; OrthoK and RGPCL = 5.70; SCL = 6.28

HCRU – health care resource utilisation, NR – not reported, OrthoK – orthokeratology, RGPCL – rigid gas-permeable contact lens, SCL – soft contact lens, TMM – traditional myopia management

*The values reported in the table have been inflated and converted to USD based on the approach described in the Methods section.

**Figure 3:**
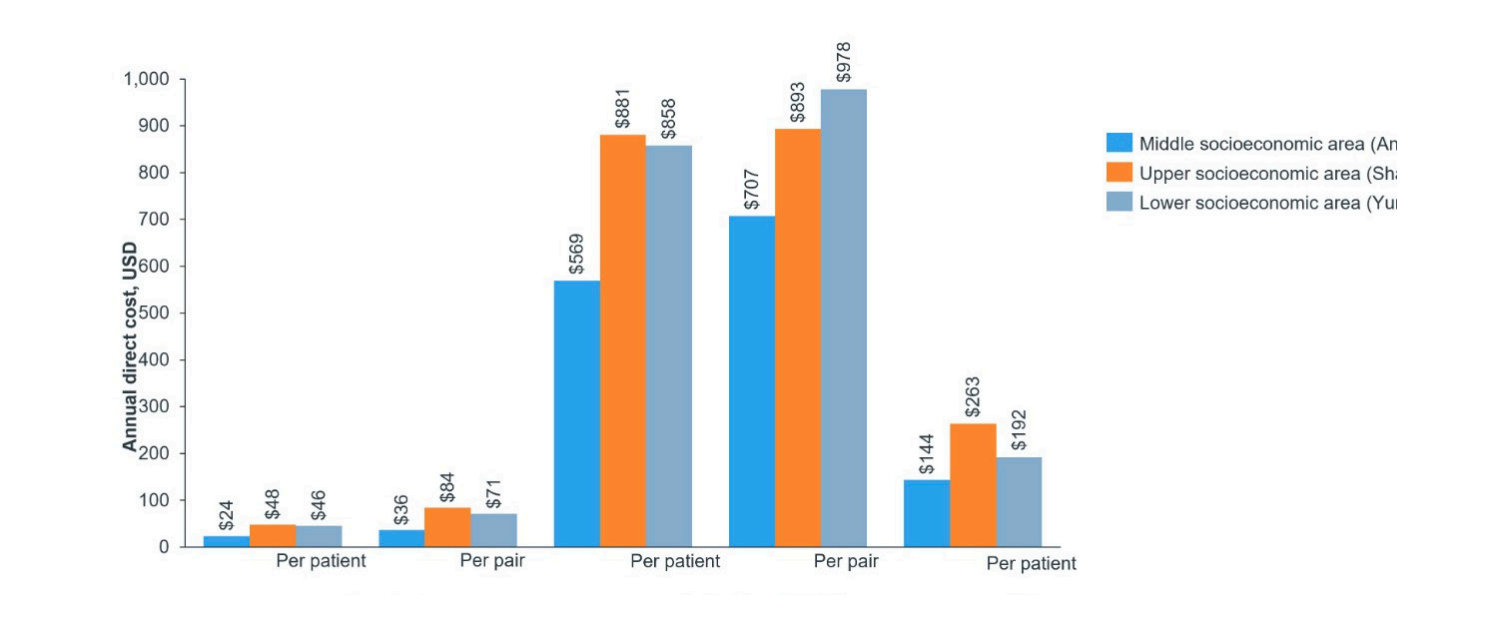
Annual direct cost of spectacles, OrthoK/RGPCL, and SCL in different socioeconomic areas of mainland China. OK/OrthoK – orthokeratology, RGPCL – rigid gas-permeable contact lens, SCL – soft contact lens.

Cost disparities were also evident in vision correction methods, with Shanghai reporting a higher annual discounted cost per pair of spectacles and annual cost per patient, followed by Yunnan and Anhui. For OrthoK and rigid gas-permeable contact lenses (RGPCL), the annual discounted costs were highest in Yunnan, followed by Shanghai and Anhui. In contrast, soft contact lenses were most expensive in Shanghai, followed by Yunnan and Anhui (**Figure 3**) [47].

A study assessing the costs of traditional myopia management in both China and Australia revealed a higher mean lifetime cost in Chinese patients 9832.94 USD (inflation adjusted) compared with 9134.10 USD (inflation adjusted) Australian patients. The lowest lifetime cost options with 3% discounting were anti-myopia spectacles (8941.27 USD; inflation adjusted) in Australia and low-dose atropine (5469.16 USD; inflation adjusted) in China (**Table 3**) [48].

#### Indirect health care costs

Indirect costs from working time loss also varied by socioeconomic area, with the highest costs reported in Shanghai, followed by Yunnan and Anhui. Since patients aged 5–20 years were mainly students and did not produce substantial social productivity, only those aged 20–50 years were included to estimate productivity loss [47].

For medical health services in hospitals, the mean annual cost of working time loss was lowest in Anhui at 5.75 USD, higher in Shanghai at 10.74 USD, and highest in Yunnan at 13.48 USD (all inflation adjusted). The myopia prevention annual cost was highest in Shanghai, followed by Anhui and Yunnan (**Table 3**) [47].

Key health care cost findings for all age groups with further details are summarised (**Table 3**; Table S8 in the **Online Supplementary Document**).

#### HCRU

All HCRU outcomes across age groups were derived from a single study conducted in China [47]. Socioeconomic status influenced HCRU and access among patients with myopia in China (**Table 3**). Notable disparities were observed in hospital visit rates among spectacle users, with higher utilisation reported in lower socioeconomic regions, such as Yunnan (28.80%), compared to middle (Anhui, 20.60%) and upper (Shanghai, 16.80%) socioeconomic areas [47]. Medical health service utilisation was highest among children aged 5–9 years across all regions, with the highest rates in Shanghai (81.50%), followed by Yunnan (73.30%) and Anhui (64.20%). These findings suggest that wealthier regions may facilitate greater access to health care services. The average interval for spectacle replacement also varied by region, with the longest duration reported in Shanghai (2.4 years), followed by Yunnan (2.3 years) and Anhui (2.1 years). Spectacle use was most prevalent among children aged 5–9 years, with the highest proportion in Shanghai (62.34%), followed by Anhui (59.35%) and Yunnan (40.11%). Usage of OrthoK (OK) and RGPCL was minimal in this age group, and no usage of SCL was reported (**Table 3**).

Among individuals aged 10–14 years, spectacles remained the most commonly used vision correction method, with Anhui reporting the highest usage rate at 82.11% [47]. Variations in the use of spectacles, OrthoK/RGPCL, and SCL across different socioeconomic regions and age groups are presented in Figure S4, Panels A–C in the **Online Supplementary Document**.

Key HCRU findings for all age groups and further details provided are summarised (**Table 3**; Table S8 in the **Online Supplementary Document**).

## DISCUSSION

This systematic review provides a broad overview of the cost-effectiveness, resource utilisation, and economic burden associated with current myopia management across all age groups and regions. Despite an initial search yielding over 3000 articles, only 20 studies were identified, thus highlighting the limited research on the economic burden of myopia [49,50]. Despite being a leading cause of visual impairment and blindness when uncorrected, myopia has been under-prioritised due to being considered as a simple refractive error along with delayed benefits of childhood interventions, which deprioritised research funding by public health authorities [51]. The recent rise in prevalence, especially in children, has prompted renewed global attention, with organisations like World Health Organisation (WHO) recognising its long-term impact [52]. The 2023 Agyekum study further highlighted this gap, noting that many economic evaluations are outdated or scarce [20].

The geographical distribution of the included studies were skewed toward East Asian countries/regions such as China, Taiwan, and Singapore [53]. This reflects early recognition of the myopia crisis due to the high prevalence and economic burden in these regions thereby facilitating greater research investments. In contrast, economic data from low-to-middle income countries (LMICs) such as South Asia, Africa and Latin America remain scarce, possibly due to historical neglect and limited funding. The rising prevalence in these regions highlight the need for country-specific studies to inform local policy and address the global research gap [47,54,55].

Paediatric vision screening is essential for early detection and prevention of myopia [56]. However, current guidelines fail to adequately address the onset and progression of myopia [57]. School-based myopia screening programmes have proven cost-effective in both developed and developing countries [58–60]. Digital strategies in China showing enhanced cost-effectiveness [34], with early interventions during preschool and early school years preventing rapid progression. Furthermore, early detection could conserve resources, enhance accessibility and affordability, and facilitate personalised management of myopia [34]. However, translating these strategies to manage myopia in LMICs requires careful consideration. High cost or technology-intensive options are challenging to execute in settings with limited resources, digital literacy, and access. Barriers such as financial constraints, limited access to care, lack of awareness, insufficient health insurance, long wait times, and transportation issues hinder implementation [57,61]. Mitigation strategies involve on-site examinations, community-based delivery using teachers or volunteers, low-cost technologies like smartphone vision tests and SMS reminders and patient follow-up care. Integrating school-based screening with free or low-cost glasses and promoting outdoor time through public health efforts, similar to Singapore can reduce the risk or delay myopia onset [10,62,63].

Pharmacological treatments, such as low-dose atropine (0.01, 0.025, 0.05%), have shown cost-effectiveness in controlling myopia progression in children and adolescents [26,31,32], with potential of improved long-term compliance and adherence due to fewer side effects [64–66]. While some Asian countries *(e.g*. Singapore, China) have already incorporated atropine into public health strategies, wider adoption in Southeast Asia and Africa could significantly reduce progression to high myopia. Non-pharmacological interventions, including outdoor activities, DIMS lenses, red-light therapy, OrthoK, and highly aspherical lenslets show promise but face scalability and equity challenges in LMIC due to high cost and reduced adherence [26,33]. Effective implementation requires alignment with local health system capacity, cultural acceptance, and affordability to ensure sustainable impact.

Managing adult myopia complications, such as mCNV is essential. Treatments for mCNV such as ranibizumab are cost-effective compared to vPDT and conbercept, although regional variations exist. Refractive correction surgeries like SMILE offer long-term cost savings through QALY gains [36], but remain financially inaccessible in LMICs due to high cost.

The current economic evaluations reveal an overreliance on cost per QALY, while DALYs, ICERs per dioptre reduction, and other endpoints were inconsistently integrated into the synthesis, thus limiting comparability. This heterogeneity underscores the need for standardised methodologies and consensus on key metrics, ideally guided by bodies like International Myopia Institute and WHO.

Costs incurred by adults for myopia management are substantial. Direct costs driven by specialist visits and vision correction exceed indirect costs [42–46], though regional and socioeconomic disparities persist [44,47,67]. For instance, in Spain, mCNV patients incurred higher direct costs but showed no significant difference in indirect costs, possibly due to limitations in cost capture methods in measuring productivity loss. [44].

In China, medical service utilisation is highest among children aged 5–9 years and declines with age; while spectacle replacement is more frequent in wealthier regions compared with middle or lower socioeconomic regions [47]. Overall, HCRU, including specialist visits, hospital visits, hospitalisation, and ER visits, is higher among adults, especially those with complications like mCNV [42,44,45].

These findings underscore the importance of evaluating myopia management costs, particularly in LMICs, where health care expenditure is a significant concern [68]. Effective strategies should prioritise early intervention, screening, and equitable access. Lack of equity in myopia services is also a pronounced challenge, with reduced accessibility in rural regions, which can be addressed through free schooling programmes, donation or low-cost spectacle schemes, and inclusion of refractive services in national insurance [69,70]. Government ownership and long-term financing are essential for sustainable scale-up, as emphasised in LMIC school eye health reviews [71]. The WHO SPECS 2030 initiative further aims to support global efforts by increasing access to corrective eyewear by 40% by 2030 [72].

In conclusion, this review highlights significant variations in myopia-related health care costs across regions, treatment types, and socioeconomic groups, reinforcing the need for standardised approaches to economic evaluations and policy planning.

A notable limitation is the identification of only 20 studies, with just 12 focusing on economic evaluations of varying quality, including some medium- or poor-quality studies. Most of the evidence was derived from Asian populations, thus limiting global applicability. Diverse economic contexts and comparators hinder comparability, and the inclusion of abstracts with limited data further restricts depth of evidence. Standardised methodologies and more region-specific analyses, especially in LMICs are needed for better applicability of findings.

## CONCLUSIONS

Myopia represents a growing global public health challenge with substantial economic implications anticipated to intensify with increasing prevalence. A multifaceted approach, including prevention, early detection, public health campaigns, improved access to affordable vision correction, and cost-effective treatments, is essential to address this burden. This study underscores the urgent need for further economic evaluations across diverse geographic regions to assimilate a global comprehensive picture. Studies focusing on head-to-head comparison of interventions would be more insightful for evaluating the various cost-effective options available. Moreover, the observed heterogeneity in outcomes emphasises the importance of establishing standardised economic modelling frameworks to enhance cross-study comparability for improved evidence-based decision-making in myopia management.

## Additional material


Online Supplementary Document

## References

[1] Recko M Stahl ED Childhood myopia: epidemiology, risk factors, and prevention. Mo Med. 2015;112:116. 25958656 PMC6170055

[2] Morgan IG Wu P-C Ostrin LA Tideman JWL Yam JC Lan W IMI risk factors for myopia. Invest Ophthalmol Vis Sci. 2021;62:3. 10.1167/iovs.62.5.3 33909035 PMC8083079

[3] Yoo SH. Myopia (nearsightedness) in children & teens. American Academy of Pediatrics; 2023. Available: https://www.healthychildren.org/English/health-issues/conditions/eyes/Pages/Myopia-Nearsightedness.aspx. Accessed: 8 December 2025.

[4] Cooper Y. With childhood myopia rates on the rise, the American Optometric Association Highlights the Importance of Ealy Intervention through Annual Eye Exams. 2019. Available: https://www.aoa.org/about-the-aoa/press-room/press-releases/myopia-rates-on-the-rise. Accessed: 8 December 2025.

[5] American Optometric Association. Optometric clinical practice guideline. Care of the Patient with Myopia. St. Louis, Missouri, USA: American Optometric Association; 2006. Available: https://www.aoa.org/AOA/Documents/Practice%20Management/Clinical%20Guidelines/Consensus-based%20guidelines/Care%20of%20Patient%20with%20Myopia.pdf. Accessed: 8 December 2025.

[6] Németh J Tapasztó B Aclimandos WA Kestelyn P Jonas JB De Faber JTH Update and guidance on management of myopia. European Society of Ophthalmology in cooperation with International Myopia Institute. Eur J Ophthalmol. 2021;31:853-83. 10.1177/1120672121998960 33673740 PMC8369912

[7] Shah VA, Lauer AK, Sundy M, Khadamy J, Cui RZ, Hsu J, et al. Pathologic Myopia (Myopic Degeneration) 2024. Available: https://eyewiki.org/Pathologic_Myopia_(Myopic_Degeneration). Accessed: 8 December 2025.

[8] American Optometric Association. Clinical Report: Myopia Management. Developed by the Evidence-based Optometry Committee. St. Louis, Missouri, USA: American Optometric Association; 2021. Available: https://global.vtivision.com/wp-content/uploads/sites/4/2023/04/Evidenced-Based-Optometry-Myopia-Clinical-Report.pdf. Accessed: 8 December 2025.

[9] Jeganathan V, Saw SM, Wong TY. Book Chapter-Ocular morbidity of pathological myopia. In: Beuerman RW, Saw SM, Tan DTH, Wong TY, ed. Myopia: Animal Models to Clinical Trials. World Scientific, Singapore Eye Research Institute: Singapore, 2010. Available: https://www.researchgate.net/publication/304818559_Myopia_Animal_Models_to_Clinical_Trials. Accessed: 8 December 2025.

[10] Sherwin JC Reacher MH Keogh RH Khawaja AP Mackey DA Foster PJ The association between time spent outdoors and myopia in children and adolescents: a systematic review and meta-analysis. Ophthalmology. 2012;119:2141–51. 10.1016/j.ophtha.2012.04.020 22809757

[11] Ohno-Matsui K Wu P-C Yamashiro K Vutipongsatorn K Fang Y Cheung CMG IMI pathologic myopia. Invest Ophthalmol Vis Sci. 2021;62:5. 10.1167/iovs.62.5.5 33909033 PMC8083114

[12] Wong TY Ferreira A Hughes R Carter G Mitchell P Epidemiology and disease burden of pathologic myopia and myopic choroidal neovascularization: an evidence-based systematic review. Am J Ophthalmol. 2014;157:9–25.e12. 10.1016/j.ajo.2013.08.010 24099276

[13] Lawrenson JG Shah R Huntjens B Downie LE Virgili G Dhakal R Interventions for myopia control in children: a living systematic review and network meta-analysis. Cochrane Database Syst Rev. 2023;2:CD014758. 36809645 10.1002/14651858.CD014758.pub2 PMC9933422

[14] Liang J Pu Y Chen J Liu M Ouyang B Jin Z Global prevalence, trend and projection of myopia in children and adolescents from 1990 to 2050: a comprehensive systematic review and meta-analysis. Br J Ophthalmol. 2025;109:362–71. 10.1136/bjo-2024-325427 39317432

[15] Wu JF Bi HS Wang SM Hu YY Wu H Sun W Refractive error, visual acuity and causes of vision loss in children in Shandong, China. The Shandong Children Eye Study. PLoS One. 2013;8:e82763. 10.1371/journal.pone.0082763 24376575 PMC3871613

[16] Koh V Yang A Saw SM Chan YH Lin ST Tan MMH Differences in prevalence of refractive errors in young Asian males in Singapore between 1996–1997 and 2009–2010. Ophthalmic Epidemiol. 2014;21:247–55. 10.3109/09286586.2014.928824 24990474

[17] Gifford KL Richdale K Kang P Aller TA Lam CS Liu YM IMI–clinical management guidelines report. Invest Ophthalmol Vis Sci. 2019;60:M184–203. 10.1167/iovs.18-25977 30817832

[18] Huang J Wen D Wang Q McAlinden C Flitcroft I Chen H Efficacy comparison of 16 interventions for myopia control in children: a network meta-analysis. Ophthalmology. 2016;123:697–708. 10.1016/j.ophtha.2015.11.010 26826749

[19] Ha A Kim SJ Shim SR Kim YK Jung JH Efficacy and safety of 8 atropine concentrations for myopia control in children: a network meta-analysis. Ophthalmology. 2022;129:322–33. 10.1016/j.ophtha.2021.10.016 34688698

[20] Agyekum S Chan PP Zhang Y Huo Z Yip BH Ip P Cost-effectiveness analysis of myopia management: A systematic review. Front Public Health. 2023;11:1093836. 10.3389/fpubh.2023.1093836 36923029 PMC10008871

[21] Cui Z Zhou W Chang Q Zhang T Wang H Meng X Cost-effectiveness of Conbercept vs. Ranibizumab for age-related macular degeneration, diabetic macular edema, and pathological myopia: population-based cohort study and Markov model. Front Med (Lausanne). 2021;8:750132. 10.3389/fmed.2021.750132 34926500 PMC8676057

[22] Sankaridurg P Tahhan N Kandel H Naduvilath T Zou H Frick KD IMI impact of myopia. Invest Ophthalmol Vis Sci. 2021;62:2. 10.1167/iovs.62.5.2 33909036 PMC8083082

[23] Wu PC Chuang MN Choi J Chen H Wu G Ohno-Matsui K Update in myopia and treatment strategy of atropine use in myopia control. Eye.(Lond). 2019;33:3–13. 10.1038/s41433-018-0139-7 29891900 PMC6328548

[24] Holy C Kulkarni K Brennan NA Predicting Costs and Disability from the Myopia Epidemic–A Worldwide Economic and Social Model. Invest Ophthalmol Vis Sci. 2019;60:5466.

[25] Vutipongsatorn K Yokoi T Ohno-Matsui K Current and emerging pharmaceutical interventions for myopia. Br J Ophthalmol. 2019;103:1539–48. 10.1136/bjophthalmol-2018-313798 31097440

[26] Agyekum S Chan PP Adjei PE Zhang Y Huo Z Yip BH Cost-effectiveness analysis of myopia progression interventions in children. JAMA Network Open. 2023;6:e2340986. 10.1001/jamanetworkopen.2023.40986 37917061 PMC10623196

[27] Inflation rate, average consumer prices Annual percent change: International Monetary Fund; 2025. Available: https://www.imf.org/external/datamapper/PCPIPCH@WEO/OEMDC/ADVEC/WEOWORLD. Accessed: 8 December 2025.

[28] Official exchange rate (LCU per US$, period average): World Bank Group; 2024. Available: https://data.worldbank.org/indicator/PA.NUS.FCRF. Accessed: 8 December 2025.

[29] Drummond MF Jefferson TO Guidelines for authors and peer reviewers of economic submissions to the BMJ. The BMJ Economic Evaluation Working Party. BMJ. 1996;313:275. 10.1136/bmj.313.7052.275 8704542 PMC2351717

[30] PMG24-Single technology appraisal and highly specialised technologies evaluation: User guide for company evidence submission template. NICE. 2024.Available: https://www.nice.org.uk/process/pmg24. Accessed: 15 December 2025.

[31] Agyekum S Zhang XJ Chan PPM Zhang Y Huo Z Yip BH Cost-Effectiveness Analysis of Atropine for Treating Myopia Progression in Children. Invest Ophthalmol Vis Sci. 2023;64:821.

[32] Hong CY Boyd M Wilson G Hong SC Photorefraction screening plus atropine treatment for myopia is cost-effective: a proof-of-concept Markov analysis. Clin Ophthalmol. 2022;16:1941–52. 10.2147/OPTH.S362342 35720738 PMC9205435

[33] Lian J McGhee S Yap M Sum R Cost-effectiveness of myopia control by use of defocus incorporated multiple segments lenses: abridged secondary publication. Hong Kong Med J. 2023;29 Suppl 7:34–6. 38148654

[34] Li R Zhang K Li S-M Zhang Y Tian J Lu Z Implementing a digital comprehensive myopia prevention and control strategy for children and adolescents in China: a cost-effectiveness analysis. Lancet Reg Health West Pac. 2023;38:100837. 10.1016/j.lanwpc.2023.100837 37520278 PMC10372367

[35] Lim MC Gazzard G Sim EL Tong L Saw SM Direct costs of myopia in Singapore. Eye (Lond). 2009;23:1086–9. 10.1038/eye.2008.225 18670466

[36] Balgos MJTD Piñero DP Canto-Cerdan M Alió del Barrio JL Alió JL Comparison of the Cost-Effectiveness of SMILE, FS-LASIK, and PRK for Myopia in a Private Eye Center in Spain. J Refract Surg. 2022;38:21–6. 10.3928/1081597X-20211007-01 35020543

[37] Liu H Li R Zhang Y Zhang K Yusufu M Liu Y Economic evaluation of combined population-based screening for multiple blindness-causing eye diseases in China: a cost-effectiveness analysis. Lancet Glob Health. 2023;11:e456–65. 10.1016/S2214-109X(22)00554-X 36702141

[38] Malcolm W Leteneux C Claxton L Taylor M Rathi H The Budget Impact of Introducing Ranibizumab in England and Wales for the Treatment of Visual Impairment Due to Choroidal Neovascularization Secondary to Pathologic Myopia. Value Health. 2013;16:A503. 10.1016/j.jval.2013.08.1149

[39] Leteneux C Claxton L Malcolm W Taylor M Rathi H Cost-Effectiveness of Ranibizumab for the Treatment of Visual Impairment Due to Choroidal Neovascularization Secondary to Pathologic Myopia in the United Kingdom. Value Health. 2013;16:A505. 10.1016/j.jval.2013.08.1162

[40] Liu J Xie S Ni W He X Ren X Wu J PDG26 Cost-effectiveness of ranibizumab versus verteporfin photodynamic therapy in the treatment of choroidal neovasuclarization secondary to pathological myopia: from chinese societal perspective. Value Health. 2019;22:S601.

[41] Zhang L Lin Z Zhang W Xuan J Cost-effectiveness analysis of conbercept versus ranibizumab for the treatment of myopic choroidal neovascularization (CNV) in China. ISPOR Europe. 2019;22 Suppl 3:S886. 10.1016/j.jval.2019.09.2566

[42] Yang MC Chen YP Tan ECH Leteneux C Chang E Chu CH Epidemiology, treatment pattern and health care utilization of myopic choroidal neovascularization: a population based study. Jpn J Ophthalmol. 2017;61:159–68. 10.1007/s10384-016-0496-3 28062929

[43] Zheng YF Pan CW Chay J Wong TY Finkelstein E Saw SM The economic cost of myopia in adults aged over 40 years in Singapore. Invest Ophthalmol Vis Sci. 2013;54:7532–7. 10.1167/iovs.13-12795 24159089

[44] Ruiz-Moreno JM Roura M en representación del grupo del estudio Mypathway Cost of myopic patients with and without myopic choroidal neovascularisation. Arch Soc Esp Oftalmol. 2016;91:265–72. 10.1016/j.oftal.2016.01.013 26900043

[45] Zaour N Heisel O Leteneux C Ma P Canadian Burden Of Choroidal Neovascularization Secondary To Pathologic Myopia: Final Results. Value Health. 2014;17:A284–5. 10.1016/j.jval.2014.03.1657

[46] Naidoo KS Fricke TR Sankaridurg P Naduvilath T Resnikoff S Frick KD Estimated global productivity loss from myopia. Invest Ophthalmol Vis Sci. 2017;58:2404.

[47] Ma Y Wen Y Zhong H Lin S Liang L Yang Y Healthcare utilization and economic burden of myopia in urban China: a nationwide cost-of-illness study. J Glob Health. 2022;12:11003. 10.7189/jogh.12.11003 35356656 PMC8934110

[48] Fricke T Sankaridurg P Naduvilath T Resnikoff S Tahhan N He M P29-Factors affecting the lifetime cost of myopia and the impact of anti myopia treatments [abstract]. Br J Ophthalmol. 2022.

[49] Bourke CM Loughman J Flitcroft DI Loskutova E O’Brien C We can’t afford to turn a blind eye to myopia. QJM. 2023;116:635–9. 10.1093/qjmed/hcz076 30911761

[50] Naidoo KS Leasher J Bourne RR Flaxman SR Jonas JB Keeffe J Global Vision Impairment and Blindness Due to Uncorrected Refractive Error, 1990-2010. Optom Vis Sci. 2016;93:227–34. 10.1097/OPX.0000000000000796 26905537

[51] Sankaridurg P Tahhan N Kandel H Naduvilath T Zou H Frick KD IMI Impact of Myopia. Invest Ophthalmol Vis Sci. 2021;62:2. 10.1167/iovs.62.5.2 33909036 PMC8083082

[52] World Health Organization. World Report on Vision. Geneva, Switzerland: World Health Organisation; 2019. Available: https://www.who.int/Publications/i/Item/9789241516570. Accessed: 25 August 2025.

[53] Naidoo KS Fricke TR Frick KD Jong M Naduvilath TJ Resnikoff S Potential lost productivity resulting from the global burden of myopia: systematic review, meta-analysis, and modeling. Ophthalmology. 2019;126:338–46. 10.1016/j.ophtha.2018.10.029 30342076

[54] Li SM Liu LR Li SY Ji YZ Fu J Wang Y Design, methodology and baseline data of a school-based cohort study in Central China: the Anyang Childhood Eye Study. Ophthalmic Epidemiol. 2013;20:348–59. 10.3109/09286586.2013.842596 24160405

[55] Jonas JB Ang M Cho P Guggenheim JA He MG Jong M IMI prevention of myopia and its progression. Invest Ophthalmol Vis Sci. 2021;62:6. 10.1167/iovs.62.5.6 33909032 PMC8083117

[56] Hutchinson AK Morse CL Hercinovic A Cruz OA Sprunger DT Repka MX Pediatric eye evaluations preferred practice pattern. Ophthalmology. 2023;130:222. 10.1016/j.ophtha.2022.10.030 36543602 PMC10680450

[57] Harewood J, Contreras M, Huang K, Leach S, Wang J. Access to Myopia Care – A Scoping Review. 2024. Available: https://nap.nationalacademies.org/resource/27734/Harewood_et_al_Access_to_Myopia_Care_-_A_Scoping_Review.pdf. Accessed: 16 December 2025.

[58] Baltussen R Naus J Limburg H Cost-effectiveness of screening and correcting refractive errors in school children in Africa, Asia, America and Europe. Health Policy. 2009;89:201–15. 10.1016/j.healthpol.2008.06.003 18621429

[59] Wang L Congdon N Hogg RE Zhang S Li M Shi Y The cost-effectiveness of alternative vision screening models among preschool children in rural China. Acta Ophthalmol. 2019;97:e419–25. 10.1111/aos.13954 30345728

[60] Frick KD Riva-Clement L Shankar MB Screening for refractive error and fitting with spectacles in rural and urban India: cost-effectiveness. Ophthalmic Epidemiol. 2009;16:378–87. 10.3109/09286580903312277 19995203

[61] Tam EK Mehravaran S Young A Duarte PB Mondino K Coleman AL Relationship between geography and refractive error from the UCLA Preschool Vision Program. Ophthalmic Epidemiol. 2021;28:32–8. 10.1080/09286586.2020.1791348 32669011

[62] Morgan IG What public policies should be developed to deal with the epidemic of myopia? Optom Vis Sci. 2016;93:1058–60. 10.1097/OPX.0000000000000980 27525535

[63] Lougheed T Myopia: the evidence for environmental factors. Environ Health Perspect. 2014;122:A12–9. 10.1289/ehp.122-A12 24380886 PMC3888556

[64] Chia A Chua W-H Cheung Y-B Wong W-L Lingham A Fong A Atropine for the treatment of childhood myopia: safety and efficacy of 0.5%, 0.1%, and 0.01% doses (Atropine for the Treatment of Myopia 2). Ophthalmology. 2012;119:347–54. 10.1016/j.ophtha.2011.07.031 21963266

[65] Yam JC Zhang XJ Zhang Y Wang YM Tang SM Li FF Three-year clinical trial of low-concentration atropine for myopia progression (LAMP) study: continued versus washout: phase 3 report. Ophthalmology. 2022;129:308–21. 10.1016/j.ophtha.2021.10.002 34627809

[66] Polling JR Kok R Tideman J Meskat B Klaver C Effectiveness study of atropine for progressive myopia in Europeans. Eye (Lond). 2016;30:998–1004. 10.1038/eye.2016.78 27101751 PMC4941076

[67] World Health Organization. Western Pacific Regional strategy for health systems based on the values of primary health care. Geneva, Switzerland: World Health Organization; 2010. Available: https://iris.who.int/server/api/core/bitstreams/168beb13-f24f-42e2-b37d-566e0d936cfa/content. Accessed: 8 December 2025.

[68] Foo LL Lanca C Wong CW Ting D Lamoureux E Saw S-M Cost of myopia correction: a systematic review. Front Med (Lausanne). 2021;8:718724. 10.3389/fmed.2021.718724 34926485 PMC8677936

[69] Keay L Friedman DS Correcting refractive error in low income countries. BMJ. 2011;343:d4793. 10.1136/bmj.d4793 21828208

[70] World Health Organization. Promising progress on eye health in African region, but challenges remain. 2024. Available: https://www.afro.who.int/news/promising-progress-eye-health-african-region-challenges-remain. Accessed: 25 August 2025.

[71] Harvey AA Morjaria P Tousignant B Priorities in school eye health in low and middle-income countries a scoping review. Eye (Lond). 2024;38:1988–2002. 10.1038/s41433-024-03032-1 38565599 PMC11269736

[72] Fletcher ER. Upwards of 800 Million People with Vision Impairments Lack Access to Eyeglasses. 2024. Available: https://healthpolicy-watch.news/upwards-of-500-million-people-with-vision-impairments-lack-access-to-eyeglasses/. Accessed: 24 February 2025.

