# Supplementary material for: Economic evaluation, resource utilisation, and associated economic burden of myopia management: a systematic literature review
Source: J Glob Health. 2025 Dec 29;15:04322. doi: 10.7189/jogh.15.04322 (PMC12746006; doi:10.7189/jogh.15.04322)
Supplement: Online Supplementary Document [file jogh-15-04322-s001.pdf]

Supplement to: de Milliano TWL, Wielink KS, Ernst F, Mulkalapalli N, Pagidgummula R, Lymperopoulou C. Economic evaluation, resource utilisation, and associated economic burden of myopia management: a systematic literature review. J Glob Health. 2025;15:04322.

Supplementary tables and figures

Tables

Supplementary Table S1. Eligibility criteria for study (PICOS)

PICOS item	Inclusion	Exclusion
Population	Patients with myopia, including children, adolescents, adults, and elderly	Patients without myopia
Intervention/Comparator	No restrictions	No restrictions
Outcomes	Health economic evaluations:<br>o Cost-effectiveness results<br>o Health benefits<br>o Utilities<br>o QALYs<br>Resource use associated with myopia treatment and complications<br>Direct medical costs (including health care and social care provision covering the cost of hospitalisation, outpatient follow-up, residential and day care, pharmaceutical interventions, and laboratory testing)<br>Indirect costs (including loss of productivity and cost of caregiver's time)<br>Societal impact (direct and indirect non-medical costs)	Publications not reporting outcomes listed in the inclusion criteria
Study design(s)	Economic evaluations:<br>Cost-effectiveness analysis<br>Cost-utility analysis<br>Cost-benefit analysis<br>Cost-consequence analysis<br>Cost-minimisation analysis<br>Cost/ resource use:<br>Resource use studies<br>Drug-utilisation studies<br>Cost analysis	Animal studies<br>Case reports<br>Editorials, notes, comments

Limits	Language: English-only studies<br>Publication years: 2009–Current<br>Geographic restrictions: No restriction	Publications in languages other than English<br>Conference abstracts published before 2022

Abbreviations: PICOS - population, intervention, comparator, outcomes, study design, QALY - quality-adjusted life-year.

Supplementary Table S2. Country and year wise inflation rates

Country of original study	Inflation rate, average consumer prices (Annual percent change)																
	**2008**	**2009**	**2010**	**2011**	**2012**	**2013**	**2014**	**2015**	**2016**	**2017**	**2018**	**2019**	**2020**	**2021**	**2022**	**2023**	**2024**
Canada	2.4	0.3	1.8	2.9	1.5	0.9	1.9	1.1	1.4	1.6	2.3	1.9	0.7	3.4	6.8	3.9	2.4
China, People's Republic of	5.8	-0.7	3.3	5.4	2.6	2.6	2	1.4	2	1.6	2.1	2.9	2.5	0.9	2	0.2	0.2
Euro area*	3.3	0.3	1.6	2.7	2.5	1.4	0.4	0.2	0.2	1.5	1.8	1.2	0.3	2.6	8.4	5.4	2.4
Hong Kong SAR	4.3	0.6	2.3	5.3	4.1	4.3	4.4	3	2.4	1.5	2.4	2.9	0.3	1.6	1.9	2.1	1.7
New Zealand	4	2.1	2.3	4	1.1	1.1	1.2	0.3	0.6	1.9	1.6	1.6	1.7	3.9	7.2	5.7	2.9
Singapore	6.6	0.6	2.8	5.3	4.6	2.4	1	-0.5	-0.5	0.6	0.4	0.6	-0.2	2.3	6.1	4.8	2.4
Spain	4.1	-0.2	2	3	2.4	1.5	-0.2	-0.6	-0.3	2	1.7	0.8	-0.3	3	8.3	3.4	2.9
Taiwan Province of China	3.5	-0.9	1	1.4	1.9	0.8	1.2	-0.3	1.4	0.6	1.4	0.6	-0.2	2	2.9	2.5	2.2
United Kingdom	3.6	2.2	3.3	4.5	2.8	2.6	1.5	0	0.7	2.7	2.5	1.8	0.9	2.6	9.1	7.3	2.5
United States	3.8	-0.3	1.6	3.1	2.1	1.5	1.6	0.1	1.3	2.1	2.4	1.8	1.3	4.7	8	4.1	3

*Euro area – Austria, Belgium, Croatia, Cyprus, Estonia, Finland, France, Germany, Greece, Ireland, Italy, Latvia, Lithuania, Luxembourg, Malta, Netherlands, Portugal, Spain, Slovak Republic, Slovenia

Abbreviations: SAR - Special Administrative Region

Source: International Monetory Fund [1]

Supplementary Table S3. Country wise exchange rates for 2024

Country of original study	Official exchange rate					
	2012	2018	2020	2021	2022	2024
Canada	0.999365	1.295818	1.341153	1.253877	1.301555	1.36936
China, People's Republic of	6.312333	6.615957	6.900767	6.448975	6.737158	7.197491
Euro area*	0.778338	0.846773	0.875506	0.845494	0.949624	0.92389
Hong Kong SAR	7.756417	7.8385	7.75725	7.77325	7.831417	7.803833
New Zealand	1.234284	1.445258	1.542058	1.4138	1.577183	1.652308
Singapore	1.249676	1.348842	1.379742	1.343483	1.378667	1.336233
Spain	0.778338	0.846773	0.875506	0.845494	0.949624	0.92389
United Kingdom	0.633047	0.749532	0.78	0.727065	0.811302	0.782415
United States	1	1	1	1	1	1

*Euro area – Austria, Belgium, Croatia, Cyprus, Estonia, Finland, France, Germany, Greece, Ireland, Italy, Latvia, Lithuania, Luxembourg, Malta, Netherlands, Portugal, Spain, Slovak Republic, Slovenia

Abbreviations: SAR - Special Administrative Region

Source: World Bank [2]

Supplementary Table S4. Drummond checklist for economic evaluations studies

Population	Paediatric & Adolescent myopia (≤18 years)					Adult myopia (>18 years)						
**Item**	**Agyekum 2023 a**	**Li 2023**	**Hong 2022**	**Agyekum 2023 b**	**Lian 2023**	**Balgos 2022**	**Cui 2021**	**Leteneux 2013**	**Liu 2019**	**Liu 2023**	**Malcolm 2013**	**Zhang 2019**
Was the research question stated?	Green	Green	Green	Green	Green	Green	Green	Green	Green	Green	Green	Green
Was the economic importance of the research question stated?	Green	Green	Green	Green	Green	Green	Green	Green	Red	Green	Red	Red
Was/were the viewpoint(s) of the analysis clearly stated and justified?	Green	Green	Green	Red	Green	Green	Green	Red	Green	Green	Green	Green
Was the rationale for choosing alternative programmes or interventions compared stated?	Red	Green	Green	Green	Green	Green	Green	Red	Red	Green	Red	Red
Were the alternatives being compared clearly described?	Green	Green	Green	Green	Green	Green	Green	Green	Green	Green	Green	Green
Was the form of economic evaluation stated?	Green	Green	Green	Green	Green	Green	Green	Green	Green	Green	Green	Green
Was the choice of form of economic evaluation justified in relation to the questions addressed?	Green	Green	Green	Green	Green	Green	Green	Green	Green	Green	Green	Green
Was/were the source(s) of effectiveness estimates used stated?	Green	Green	Green	Green	Red	Green	Green	Green	Green	Green	Red	Green
Were details of the design and results of the effectiveness study given (if based on a single study)?	Blue	Red	Green	Green	Red	Red	Green	Red	Red	Red	Red	Red
Were details of the methods of synthesis or meta-analysis of estimates given (if based on an overview of a number of effectiveness studies)?	Red	Blue	Green	Green	Blue	Blue	Blue	Blue	Blue	Blue	Blue	Blue
Were the primary outcome measure(s) for the economic evaluation clearly stated?	Green	Green	Green	Green	Green	Green	Green	Green	Green	Green	Green	Green
Were the methods used to value health states and other benefits stated?	Green	Green	Green	Red	Green	Green	Green	Green	Green	Green	Blue	Green
Were the details of the subjects from whom valuations were obtained given?	Red	Green	Red	Red	Red	Red	Green	Red	Red	Red	Red	Red
Were productivity changes (if included) reported separately?	Red	Green	Blue	Red	Red	Red	Blue	Blue	Blue	Green	Blue	Blue
Was the relevance of productivity changes to the study question discussed?	Green	Green	Blue	Red	Red	Red	Blue	Blue	Blue	Green	Blue	Blue
Were quantities of resources reported separately from their unit cost?	Red	Green	Green	Red	Red	Red	Red	Red	Red	Red	Red	Red
Were the methods for the estimation of quantities and unit costs described?	Red	Green	Green	Red	Green	Red	Red	Red	Red	Red	Red	Red
Were currency and price data recorded?	Green	Green	Green	Green	Red	Green	Red	Red	Red	Red	Red	Red
Were details of price adjustments for inflation or currency conversion given?	Green	Red	Green	Red	Red	Red	Red	Red	Red	Green	Red	Red
Were details of any model used given?	Green	Green	Green	Green	Red	Green	Green	Red	Green	Green	Green	Green
Was there a justification for the choice of model used and the key parameters on which it was based?	Green	Green	Green	Green	Green	Green	Green	Red	Red	Green	Red	Red
Was the time horizon of cost and benefits stated?	Green	Green	Green	Green	Green	Green	Green	Green	Green	Green	Green	Green

Population	Paediatric & Adolescent myopia (≤18 years)					Adult myopia (>18 years)						
**Item**	**Agyekum 2023 a**	**Li 2023**	**Hong 2022**	**Agyekum 2023 b**	**Lian 2023**	**Balgos 2022**	**Cui 2021**	**Leteneux 2013**	**Liu 2019**	**Liu 2023**	**Malcolm 2013**	**Zhang 2019**
Was the discount rate stated?	Yes	Yes	Yes	No	Yes	Yes	No	Yes	No	Yes	No	No
Was the choice of rate justified?	Yes	No	No	No	No	No	Not applicable	No	Not applicable	Yes	Not applicable	Not applicable
Was an explanation given if cost or benefits were not discounted?	No	Not applicable	Not applicable	No	Not applicable	Yes	No	Not applicable	No	Not applicable	No	No
Were the details of statistical test(s) and confidence intervals given for stochastic data?	No	Yes	No	No	Yes	No	No	No	No	Yes	No	No
Was the approach to sensitivity analysis described?	Yes	Yes	Yes	Yes	Yes	Yes	Yes	No	Yes	Yes	Not applicable	Yes
Was the choice of variables for sensitivity analysis justified?	No	Yes	Yes	Yes	No	No	Yes	No	No	Yes	Not applicable	No
Were the ranges over which the parameters were varied stated?	Yes	Yes	No	Yes	No	Yes	Yes	No	No	Yes	Not applicable	No
Were relevant alternatives compared? (That is, were appropriate comparisons made when conducting the incremental analysis?)	Yes	Yes	Yes	Yes	Yes	Yes	Yes	No	Yes	Yes	Yes	Yes
Was an incremental analysis reported?	Yes	Yes	Yes	Yes	Yes	Yes	Yes	No	Yes	Yes	Yes	No
Were major outcomes presented in a disaggregated as well as aggregated form?	No	Yes	No	No	No	No	No	No	No	Yes	No	No
Was the answer to the study question given?	Yes	Yes	Yes	Yes	Yes	Yes	Yes	Yes	Yes	Yes	Yes	Yes
Did conclusions follow from the data reported?	Yes	Yes	Yes	Yes	Yes	Yes	Yes	Yes	Yes	Yes	Yes	Yes
Were conclusions accompanied by the appropriate caveats?	Yes	Yes	Yes	Yes	Yes	Yes	Yes	No	No	Yes	No	No

No   Not clear   Yes   Not applicable

Supplementary Table S5. Characteristics of economic evaluation studies

Reference	Study population	Intervention	Comparator	Region, country	Type of economic analysis	Modelling approach	Currency, cost year	Time horizon	Analysis perspective	Discount rate	Study outcomes
**Paediatric & adolescent myopia (≤18 years)**											
Li 2023 [3]	Childhood myopia	DCMPC	TMPC, SMSP	Mainland China	CEA & CUA	Markov	USD, NR	12 years	Societal perspective	Cost and outcome: 3.5%	1) QALY; 2) DALY; 3) ICUR; 4) ICER
Agyekum 2023[a] [4]	Childhood myopia	Atropine 0.05%, Atropine 0.01%, DIMS, Daily disposable SCL, MSCLs, RGPCLs, BSLs, HALs, PALs, Outdoor, Red-light therapy, OrthoK	SVL	Hong Kong	CEA	Markov	USD, 2022	5 years	Societal perspective	Cost: 3%	1) Cost; 2) Change in SE and AL over 1 year; 3) ICER
Agyekum 2023[b] [5]	Childhood myopia	Atropine 0.05%, Atropine 0.025%, Atropine 0.01%	NR	Hong Kong	CEA	Markov	USD, NR	20 years	Societal perspective	NR	ICER
Lian 2023 [6]	Childhood myopia	DIMS	No myopia control	Hong Kong	CEA	Markov	HKD, NR	Lifetime	Public health care/ Patients/ Society perspectives	Cost and outcome: 3.5%	1) Incremental costs; 2) Incremental QALYs gained; 3) ICER
Hong 2022 [7]	Childhood myopia	Photorefraction Screening + Atropine	Usual care	New Zealand	CEA	Markov	NZD, 2021	Lifetime	Societal perspective	Cost and outcome: 3%	1) ICER (NZD/QALY); 2) Effect (QALYs per person screened); 3) Excess lifetime cost (NZD) per 100,000 screened; 4) Incremental cost (NZD) per person; 5) QALYs gained per 100,000 screened; 6) Blindness prevented per 100,000 screened; 7) Number needed to screen to prevent one blindness
**Adult myopia (>18 years)**											
Cui 2021 [8]	PM	Conbercept	Ranibizumab	Mainland China	CEA	Markov	CNY, NR	10 years	Payer perspective	Cost and outcome: 3.5%	1) Costs; 2) QALYs; 3) ICER
Liu 2019 [9]	CNV secondary to PM	Ranibizumab	vPDT	Mainland China	CEA	Markov	CNY, NR	Lifetime	Societal perspective	NR	1) Costs; 2) QALYs; 3) ICER

Reference	Study population	Intervention	Comparator	Region, country	Type of economic analysis	Modelling approach	Currency, cost year	Time horizon	Analysis perspective	Discount rate	Study outcomes
Liu 2023 [10]	Multiple eye diseases (AMD, glaucoma, diabetic retinopathy, cataracts, and PM)	Population-based screening programmes	No screening	China	CEA & CUA	Markov	USD, 2021	30 years	Societal perspective	Cost and outcome: 3.5%	1) ICUR using QALY; 2) ICER
Zhang 2019 [11]	mCNV	Conbercept	Ranibizumab	China	CEA	Markov	CNY, NR	Lifetime	Payer perspective	NR	1) Total cost; 2) QALYs; 3) Probability of being cost-effective
Leteneux 2013 [12]	CNV secondary to PM	Ranibizumab	vPDT	UK	CEA	Markov	GBP, NR	Lifetime	Health care provider perspective	Cost and outcome: 3.5%	1) Mean lifetime cost of ranibizumab treatment; 2) QALYs; 3) Utility values; 4) WTP of £20,000 per QALY
Balgos 2022 [13]	Myopia	SMILE, FS-LASIK, PRK	NR	Spain	CEA	Decision tree	EUR, 2020	30 years	Payer and health care sector (eye centre) perspective	Cost and outcome: 3%	**Payer perspective:** 1) Average weighted costs; 2) Average weighted utility values; 3) QALY; 4) Cost per QALY (ICER)<br>**Health care systems perspective:** 1) The outcomes obtained were the income from the procedures (in euros); 2) Costs (in euros) to the health centre
Malcolm 2013 [14]	CNV secondary to PM	Ranibizumab	vPDT	England and Wales	BIA	Open cohort model	GBP, NR	5 years	NHS perspective	NR	1) Eligible patients for treatment per year; 2) Patients treated with ranibizumab and verteporfin photodynamic therapy; 3) Costs per year; 4) Cost savings per year

Abbreviations: AL - axial length, AMD, age-related macular degeneration, BIA - budget impact analysis, BSL - bifocal soft lens, CEA - cost-effectiveness analysis, CNV - choroidal neovascularisation, CNY - Chinese yuan, CUA - cost-utility analysis, DALY - disability-adjusted life-year, DCMPC - digital comprehensive myopia prevention and control, DIMS - defocus incorporated multiple segments spectacle lens, EUR - euro, FS-LASIK - femtosecond laser-assisted in situ keratomileusis, GBP – great British pound sterling, HAL - highly aspherical lenslet, HKD - Hong Kong dollar, ICER - incremental cost-effectiveness ratio, ICUR - incremental cost-utility ratio, mCNV - myopic choroidal neovascularisation, MSCL - multifocal soft contact lens, NHS - national health service, NR - not reported, NZD - New Zealand dollar, OrthoK - orthokeratology, PAL - progressive addition lens, PM - pathological myopia, PRK - photorefractive keratectomy, QALY - quality-adjusted life-year, RGPCL – rigid gas-permeable contact lens, SE - spherical equivalent, SCL - soft contact lens, SMILE - small incision lenticule extraction, SMSP - school-based myopia screening, SVL - single vision lens, TMPC - traditional myopia-prevention and control, UK – United Kingdom; USD - United States dollar, vPDT - verteporfin photodynamic therapy, WTP - willingness to pay.

Supplementary Table S6. Economic evaluations and health care costs findings for paediatric and adolescent populations

Economic evaluations											
**Reference**	**Outcomes**	**Country/Region**	**Intervention**	**Intervention Costs, mean**	**Intervention QALYs**	**Comparator**	**Comparator Costs, mean**	**Comparator QALYs**	**Incremental costs**	**Incremental QALYs**	**Cost-effectiveness/Cost-utility**
Li 2023 [3]	CEA & CUA	Mainland China	**Rural setting**								
			DCMP	$324[*]	9.59448[*]	TMPC	$218[*]	9.58509[*]	$10,600,000[#]	939[#]	ICUR: $11,301
			TMPC	$218[*]	9.58509[*]	SMSP	$171[*]	9.57764[*]	$4,700,000[#]	745[#]	ICUR: $6,309
			DCMP	$324[*]	0.05905[**]	TMPC	$218[*]	0.06189[**]	$10,600,000[#]	284[##]	ICER: $37,446
			TMPC	$218[*]	0.06189[**]	SMSP	$171[*]	0.06562[**]	$4,700,000[#]	373[##]	ICER: $12,588
			**Urban setting**								
			DCMP	$819[*]	9.57964[*]	TMPC	$729[*]	9.57119[*]	$9,000,000[#]	845[#]	ICUR: $10,707
			TMPC	$729[*]	9.57119[*]	SMSP	$607[*]	9.56321[*]	$12,200,000[#]	798[#]	ICUR: $15,271
			DCMP	$819[*]	0.05670[**]	TMPC	$218[*]	0.06189[**]	$10,600,000[#]	216[##]	ICER: $41,814
			TMPC	$729[*]	0.05886[**]	SMSP	$171[*]	0.06552[**]	$4,700,000[#]	666[##]	ICER: $18,295
Agyekum 2023 [4]	CEA	Hong Kong	**Spherical equivalent refraction**								
			Atropine 0.05%	HK$ (US$): 43,615 (6,193)	3.42	SVLs	HK$ (US$): 34,320 (4,372)	-4.86	HK$ (US$): 14,303 (1822)	8.28	ICER HK$ (US$): 1,727 (220) /reduction[c]
			Atropine 0.01%	HK$ (US$):49,070 (6,251)	1.98	SVLs	HK$ (US$): 34,320 (4,372)	-4.86	HK$ (US$): 14,750 (1,879)	6.84	ICER HK$ (US$):2,159 (275)/reduction
			DIMS	HK$ (US$):63,036 (8,030)	1.68	SVLs	HK$ (US$): 34,320 (4,372)	-4.86	HK$ (US$):28,715 (3,658)	6.54	ICER HK$ (US$): 4,388 (559)/reduction
			Daily disposable CLs	HK$ (US$):93,588 (11,922)	1.50	SVLs	HK$ (US$): 34,320 (4,372)	-4.86	HK$ (US$): 56,268 (7,550)	6.36	ICER HK$ (US$): 9,318 (1,187)/reduction
			MSCLs	HK$ (US$): 88,313 (11,250)	1.56	SVLs	HK$ (US$): 34,320 (4,372)	-4.86	HK$ (US$): 54,000 (6,879)	6.42	ICER HK$ (US$): 8,407 (1,071)/reduction
			RGPCLs	HK$ (US$): 75,517 (9,620)	1.80	SVLs	HK$ (US$): 34,320 (4,372)	-4.86	HK$ (US$): 41,197 (5,248)	6.66	ICER HK$ (US$): 6,186 (788)/reduction

			BSLs	HK$ (US$): 46,621 (5,939)	-0.40	SVLs	HK$ (US$): 34,320 (4,372)	-4.86	HK$ (US$): 12,301 (1,567)	4.46	ICER HK$ (US$): 2,763 (352)/reduction
			HALs	HK$ (US$): 58,592 (7,464)	2.04	SVLs	HK$ (US$): 34,320 (4,372)	-4.86	HK$ (US$): 24,272 (3,092)	6.90	ICER HK$ (US$): 3,517 (448)/reduction
			PALs	HK$ (US$): 66,858 (8,517)	0.78	SVLs	HK$ (US$): 34,320 (4,372)	-4.86	HK$ (US$): 32,538 (4,145)	5.64	ICER HK$ (US$): 5,770 (735)/reduction
			Outdoor	HK$ (US$): 34,108 (4,345)	0.96	SVLs	HK$ (US$): 34,320 (4,372)	-4.86	HK$ (US$): -204 (-26)	5.82	ICER HK$ (US$): -39 (-5) /reduction[c]
			Red-light therapy	HK$ (US$):90,102 (11,478)	3.54	SVLs	HK$ (US$): 34,320 (4,372)	-4.86	HK$ (US$):55,782 (7,106)	8.40	ICER HK$ (US$): 6,641 (846)/reduction[c]
			**Axial length**								
			Atropine 0.05%	HK$ (US$): 43,615 (6,193)	1.80	SVLs	HK$ (US$): 34,320 (4,372)	-2.46	HK$ (US$): 14,303 (1822)	4.26	ICER HK$ (US$): 3,360 (428) /reduction[c]
			Atropine 0.01%	HK$ (US$):49,070 (6,251)	1.02	SVLs	HK$ (US$): 34,320 (4,372)	-2.46	HK$ (US$): 14,750 (1,879)	3.48	ICER HK$ (US$):4,239 (540)/reduction
			DIMS	HK$ (US$):63,036 (8,030)	0.96	SVLs	HK$ (US$): 34,320 (4,372)	-2.46	HK$ (US$):28,715 (3,658)	3.42	ICER HK$ (US$): 8,400 (1,070)/reduction
			Daily disposable CLs	HK$ (US$):93,588 (11,922)	0.72	SVLs	HK$ (US$): 34,320 (4,372)	-2.46	HK$ (US$): 56,268 (7,550)	3.18	ICER HK$ (US$): 18,636 (2,374)/reduction
			MSCLs	HK$ (US$): 88,313 (11,250)	0.39	SVLs	HK$ (US$): 34,320 (4,372)	-2.46	HK$ (US$): 54,000 (6,879)	2.85	ICER HK$ (US$): 18,950 (2,414)/reduction
			RGPCLs	HK$ (US$): 75,517 (9,620)	0.32	SVLs	HK$ (US$): 34,320 (4,372)	-2.46	HK$ (US$): 41,197 (5,248)	2.78	ICER HK$ (US$): 14,821 (1,888)/reduction
			BSLs	HK$ (US$): 46,621 (5,939)	0.40	SVLs	HK$ (US$): 34,320 (4,372)	-2.46	HK$ (US$): 12,301 (1,567)	2.86	ICER HK$ (US$): 4,302 (548)/reduction
			HALs	HK$ (US$): 58,592 (7,464)	1.02	SVLs	HK$ (US$): 34,320 (4,372)	-2.46	HK$ (US$): 24,272 (3,092)	3.48	ICER HK$ (US$): 6,979 (889)/reduction
			PALs	HK$ (US$): 66,858 (8,517)	0.90	SVLs	HK$ (US$): 34,320 (4,372)	-2.46	HK$ (US$): 32,538 (4,145)	3.36	ICER HK$ (US$): 9,687 (1,234)/reduction

			Outdoor	HK$ (US$): 34,108 (4,345)	0.60	SVLs	HK$ (US$): 34,320 (4,372)	-2.46	HK$ (US$): -204 (-26)	3.06	ICER HK$ (US$): -63 (-8) /reduction[c]
			Red-light therapy	HK$ (US$):90,102 (11,478)	1.80	SVLs	HK$ (US$): 34,320 (4,372)	-2.46	HK$ (US$):55,782 (7,106)	4.26	ICER HK$ (US$): 3,360 (428)/reduction[c]
			OrthoK	HK$ (US$): 120,474 (15,347)	2.16	SVLs	HK$ (US$): 34,320 (4,372)	-2.46	HK$ (US$):86,154 (10,975)	4.62	ICER HK$ (US$):18,652 (2,376)[c]
Agyekum 2023 [5]	CEA	Hong Kong	Atropine 0.05%	NR	NR	NR	NR	NR	NR	NR	ICER: $119/SE reduction
			Atropine 0.025%								ICER: $186/SE reduction
			Atropine 0.01%								ICER: $247/SE reduction
Lian 2023 [6]	CEA	Hong Kong	DIMS	HK$57,850	72.78	No myopia control	HK$47,937	72.59	HK$9,913	0.19	ICER (3.5% discount rate on costs): HK$52,792 per QALY gained<br>ICER (3.5% discount rate on QALYs): HK$181,794 per QALY gained
Hong 2022 [7]	CEA	New Zealand	Photorefraction screening + atropine 0.01%	NR	0.0129*	Usual care	NR	NR	NZ$17.70*	QALYs gained: 1290#<br>Blindness prevented: 7#	ICER: NZ$1,590.42 (95% CI 1,390.10–1,790.74) (per QALYs)

**Health care costs**								
**Reference**	**Country/Region**	**Currency**	**Cost year**	**Subgroups**	**Sample size**	**Description**	**Unit**	**Value**
**Direct costs**								
Lim 2009 [15]	Singapore	SGD (1 USD = 1.5 SGD)	NR	Childhood myopia	337	Annual cost, SGD or USD	Mean (95% CI)	221.70 (186.50–258.10) or 147.80 (124.30–172.10)
					337	Annual cost per subject, SGD or USD	Median (SD)	125.00 (NR) or 83.30 (NR)

					334	Spectacles, cost per pair, SGD or USD	Mean (95% CI)<br>Mean	123.20 (116.60–129.80) or 82.1 (77.80–86.50)
					60	Annual cost of contact lenses, SGD or USD		567.10 (422.20–712.00) or 378.10 (281.40–474.60)
					293	Cost of optometrist visit, SGD		126.10 (119.30–133.00)
					300	Transport cost, per subject, SGD or USD		9.59 or 6.39

Abbreviations: AL - axial length, BSL - bifocal soft lens, CEA - cost-effectiveness analysis, CI - confidence interval, CL - contact lens, CUA - cost-utility analysis, DALY – disability-adjusted life-years, DCMP - digital comprehensive myopia prevention, DIMS - defocus incorporated multiple segments spectacle lens, HAL - highly aspherical lenslet, HK$ - Hong Kong dollar, ICER - incremental cost -effectiveness ratio, ICUR – incremental cost utility analysis, MSCL - multifocal soft contact lenses, NR - not reported, NZ$ - New Zealand dollars, OrthoK - orthokeratology, PAL - progressive addition lens, QALY - quality-adjusted life-year, RGPCL - rigid gas-permeable contact lens, SD - standard deviation, SE - spherical equivalent, SER - spherical equivalent refraction, SGD - Singapore dollar, SMSP - school-based myopia screening, SVL - single vision lens, TMPC - traditional multi-prevention and control, USD - United States dollar.q**Note:** None of the studies reported data on the life-years gained or incremental life-years. [*]per person. [#]per 100,000 people. [**]DALYs per person. [##]DALY averted per 100,000 people.

[a] and [b] in the Agyekum study reflect the same author for different papers.

[c] Dominant strategies.

In Agyekum 2023 [a]: Unit of measure is diopters for spherical equivalent refraction and millimetres for axial length.

Note: Values reported are in the currency reported in the original articles (unconverted and uninflated)

Supplementary Table S7. Economic evaluations, costs, and HCRU findings for adult population

Economic evaluations												
**Reference**	**Study population**	**Outcomes**	**Country/Region**	**Intervention**	**Intervention Costs, mean**	**Intervention QALYs**	**Comparator**	**Comparator Costs, mean**	**Comparator QALYs**	**Incremental costs**	**Incremental QALYs**	**Cost-effectiveness/Cost-utility**
Zhang 2019 [11]	mCNV	CEA	Mainland China	Conbercept (Scenario 1[#])	CNY 180,207	9.86	Ranibizumab (Scenario 1[#])	CNY 175,955	9.83	NR	NR	Conbercept was cost-effective under the threshold of three times GDP per capita.
				Conbercept (Scenario 2[#])	CNY 163,987	9.86	Ranibizumab (Scenario 2[#])	CNY 164,923	9.83			Total costs of conbercept were lower than ranibizumab, making conbercept a dominant choice.
Liu 2019 [9]	CNV secondary to PM	CEA	Mainland China	Ranibizumab	CNY 73,312	10.72	vPDT	CNY 59,347	10.29	NR	NR	ICER: CNY 32,477 (cost per QALYs gained)
Cui 2021 [8]	PM	CEA	Mainland China	Ranibizumab	117,198.41, RMB	7.499	Conbercept	106,587.01, RMB	7.528	-10,611.40, RMB[^^]	0.029 (per treatment pair)	ICER: -373,185.39 (cost per QALYs gained)[^^]
Liu 2023 [10]	Multiple eye diseases (AMD, glaucoma, diabetic retinopathy, cataracts, and PM)	CEA & CUA	Mainland China	**Rural setting**								
				Non-telemedicine screening	$2,268[*]	15.01874[*]	No screening	$2,189[*]	14.98733[*]	$7,832,049[##]	3141[##]	ICUR: $2,494 (1,130 to 2,716)
				Non-AI telemedicine screening	$2,263[*]	15.01898[*]		$2,189[*]	14.98733[*]	$7,361,727[##]	3165[##]	ICUR: $2,326 (1,064 to 2,538)
				AI telemedicine screening	$2,059[*]	15.01936[*]		$2,189[*]	14.98733[*]	-$13,004,906[##]	3203[##]	ICUR: Dominating
				Non-telemedicine screening	$2,268[*]	0.49333 (years of blindness[*])		$2,189[*]	0.49961 (years of blindness[*])	$7,832,049[##]	627 (years of blindness avoided[##])	ICER: $12,487 (8,773 to 18,791)
				Non-AI telemedicine screening	$2,263[*]	0.49335 (years of blindness[*])		$2,189[*]	0.49961 (years of blindness[*])	$7,361,727[##]	626 (years of blindness avoided[##])	ICER: $11,766 (8,200 to 18,000)

				AI telemedicine screening	$2,059[*]	0.49296 (years of blindness[*])		$2,189[*]	0.49961 (years of blindness[*])	-$13,004,906[##]	664 (years of blindness avoided[##])	ICER: Dominating
				**Urban setting**								
				Non-telemedicine screening	$3,075[*]	14.53944[*]	No screening	$2,985[*]	14.39514[*]	$9,004,056[##]	14430[##]	ICUR: $624 (395 to 907)
				Non-AI telemedicine screening	$3,081[*]	14.56073[*]		$2,985[*]	14.39514[*]	$9,614,636[##]	16559[##]	ICUR: $581 (368 to 864)
				AI telemedicine screening	$3,027[*]	14.56473[*]		$2,985[*]	14.39514[*]	$4,145,752[##]	16959[##]	ICUR: $244 (-315 to 1,073)
				Non-telemedicine screening	$3,075[*]	0.55790 (years of blindness[*])		$2,985[*]	0.57032 (years of blindness[*])	$9,004,056[##]	1242 (years of blindness avoided[##])	ICER: $7,251 (4,238 to 13,501)
				Non-AI telemedicine screening	$3,081[*]	0.55643 (years of blindness[*])		$2,985[*]	0.57032 (years of blindness[*])	$9,614,636[##]	1389 (years of blindness avoided[##])	ICER: $6,920 (3,926 to 13,231)
				AI telemedicine screening	$3,027[*]	0.55417 (years of blindness[*])		$2,985[*]	0.57032 (years of blindness[*])	$4,145,752[##]	1615 (years of blindness avoided[##])	ICER: $2,567 (-4,111 to 15,389)
Leteneux 2013 [12]	CNV secondary to PM	CEA	United Kingdom	Ranibizumab	NR	NR	vPDT	NR	NR	The mean lifetime cost of ranibizumab is slightly lower than vPDT	0.43 (relative gain) Ranibizumab lifetime QALYs is higher than vPDT	Ranibizumab dominated vPDT
Balgos 2022 [13]	Adult myopia	CEA	Spain	SMILE	24	Average weighted, €355.45	NR	NR	NR	NR	NR	Cost per QALY: €13.98
				PRK	24	Average weighted, €443	NR	NR	NR	NR	NR	Cost per QALY: €18.46
				FS-LASIK	23.1	Average weighted, €346.96	NR	NR	NR	NR	NR	Cost per QALY: €15.02

Health care costs									
**Reference**	**Population**	**Country/Region**	**Currency**	**Cost year**	**Subgroups**	**Sample size**	**Description**	**Unit**	**Value**
**Direct costs**									
Yang 2017 [16]	mCNV	Taiwan	USD converted from TWD (1 USD = 30.3 TWD)	2011	vPDT: mCNV patients at 1st-year treatment visit[¶]	198	Verteporfin	Mean (SD)	1,384.20 (NR)
							Laser photodynamic therapy		198.00 (NR)
							Total, per visit		16.22 (11.49); p = 0.0002 vs. IVI
					vPDT: mCNV patients at 2nd-year treatment visit[¶]	18	Total, per visit		12.09 (6.05)
					vPDT: mCNV patients at 1st-year monitoring visit[¶¶]	111			179.63 (154.29)
					vPDT: mCNV patients at 2nd-year monitoring visit[¶¶]	45			135.82 (144.29)
					IVI: mCNV patients at 1st-year treatment visit[¶]	776	Injection		49.00 (NR)
							Bevacizumab		301.10 (NR)
							Ranibizumab		1,091.90 (NR)
							Total, per visit		57.18 (113.37)
					IVI: mCNV patients at 2nd-year treatment visit[¶]	60	Total, per visit		41.03 (45.97)
					IVI: mCNV patients at 1st-year monitoring visit[¶¶]	603			188.21 (341.20)
					IVI: mCNV patients at 2nd-year monitoring visit[¶¶]	232			149.61 (18.50)
					Laser photocoagulation: mCNV patients at 1st-year treatment visit[¶]	109			28.72 (39.94)
					Laser photocoagulation: mCNV patients at 2nd-year treatment visit[¶]	6			20.69 (13.12)
					Laser photocoagulation: mCNV patients at 1st-year monitoring visit[¶¶]	54			175.27 (339.69)
					Laser photocoagulation: mCNV patients at 2nd-year monitoring visit[¶¶]	17			146.72 (190.06)
Zheng 2013 [17]	Myopia	Singapore	USD converted	2011	Myopia	113	Total annual cost (SGD), PPPY	Mean (SD)	900.89 (1,436.87)

			from SGD (1 USD = 1.27 SGD)				Total lifetime cost (SGD), per capita		21,616.00 (NR)
						103	Annual costs of optometry visits and use of spectacles & contact lenses (SGD), PPPY		587.86 (697.74)
							Lifetime costs of optometry visits and use of spectacles & contact lenses (SGD), per capita		17,105 (NR)
						7	Annual cost of LASIK surgery (SGD), PPPY		4,891.71 (1,602.20)
							Lifetime cost of LASIK surgery (SGD), per capita		4,891.71 (NR)
						3	Annual cost of complications due to LASIK/contact lenses (SGD), PPPY		33.33 (57.74)
							Lifetime cost of complications due to LASIK/contact lenses (SGD), per capita		33.33 (NR)
						1	Annual cost of pathologic myopic lesions (SGD), PPPY		1,010.00 (NR)
							Lifetime cost of pathologic myopic lesions (SGD), per capita		1,010.00 (NR)
					20-24 years [Saw et al. IOVS 2012, ARVO E-Abstract 2301]	204,000	Total annual treatment cost (SGD)		45,288,000.00 (NR)
					25-29 years [SP2 (unpublished)]	236,340			52,467,480.00 (NR)
					30-34 years [SP2 (unpublished)]	267,750			59,440,500.00 (NR)
					35-39 years [SP2 (unpublished)]	284,580			63,176,760.00 (NR)
					40-44 years [SEED]	137,970			131,347,440.00 (NR)
					45-49 years [SEED]	145,800			138,801,600.00 (NR)
					50-54 years [SEED]	107,835			133,284,060.00 (NR)

					55-59 years SEED	91,245			112,778,820.00 (NR)
					60-64 years SEED	62,460			47,656,980.00 (NR)
					65-69 years SEED	33,720			25,728,360.00 (NR)
					70-74 years SEED	30,000			22,890,000.00 (NR)
					75-79 years SEED	20,070			15,313,410.00 (NR)
					>80 years SEED	21,990			16,778,370.00 (NR)
Ruiz-Moreno 2016 [18]	mCNV or myopia without mCNV	Spain	EUR	2014	Group I: mCNV	137	Annual medical	Mean (95% CI)	1,985.10 (172.30–2,197.80)
							Annual non-medical		256.40 (11.00–523.80)
							Total cost		2,241.50 (1,897.6–2,585.30)
							Annual medical transportation (ambulances), per patient	Mean	3.70
					Group II: Myopia without mCNV	48	Annual medical, per patient	Mean (95% CI)	356.4 (250.50–497.80)
							Annual non-medical		18.60 (11.00–26.30)
							Total cost		375.00 (269.00–498.60)
							Annual medical transportation (ambulances), per patient	Mean	0.00
					Total average of all regions	NR	Annual medical	Mean	1,562.50
							Annual non-medical		194.70
							Total		1,757.20
					Centre-Interior region	NR	Annual medical		1,977.10

							Annual non-medical		419.40
							Total		2,396.40
					South region	NR	Annual medical		1,447.40
							Annual non-medical		125.40
							Total		1,572.70
					East-Levante region	NR	Annual medical		1,425.60
							Annual non-medical		48.80
							Total		1,474.30
					North-Cantabrian region	NR	Annual medical		1,138.80
							Annual non-medical		17.90
							Total		1,156.70
Zaour 2014 [19]	CNV secondary to PM, or mCNV	Canada	NR	NR	CNV secondary to PM, or mCNV	98	Complication: Mild vision loss, per patient	Mean (SD)	2,964.00 (NR)
							Complication: Moderate vision loss, per patient		3,595.00 (NR)
							Complication: Severe vision loss		4,060.00 (NR)
							mCNV-related cost		3,433.00 (NR)
**Indirect costs**									
Ruiz-Moreno 2016 [18]	mCNV or myopia without mCNV	Spain	EUR	2014	Group I: mCNV	137	Annual indirect cost, per patient	Mean (95% CI)	4,102.90 (362.70–7,843.20)
					Group II: Myopia without mCNV	48	Annual indirect cost, per patient	Mean (95% CI)	4,118 (2,621.80–11,215.90)
Naidoo 2017 [20]	Myopia and MMD	Global	USD	NR	Visual impairment associated with uncorrected myopia	NR	Global economic productivity loss cost (including informal carer cost), billion per year	NR (95% CI)	389.50 (142.00–960.90)
							Informal carer cost, billions per year		27.20 (10.00–66.00)
					Visual impairment associated with MMD		Global economic productivity loss cost (including informal carer cost), billions per year		3.00 (1.10–10.30)
							Informal carer cost, billion per year		0.20 (0.10–0.80)

					Visual impairment associated with uncorrected myopia and MMD		Overall global economic productivity loss, billion per year		392.40 (143.00–970.80)
					Visual impairment associated with uncorrected myopia and MMD		Overall informal carer cost, billion per year		27.40 (10.00–66.40)
**HCRU**									
**Reference**	**Population**	**Country/Region**	**Currency**	**Cost year**	**Subgroups**	**Sample size**	**Description**	**Unit**	**Value**
Yang 2017 [16]	mCNV	Taiwan	USD converted from TWD (1 USD = 30.3 TWD)	2011	vPDT	198, 18	Number of treatment visits at 1st year, Number of treatment visits at 2nd year	Mean (SD)	1.22 (0.51), 1.06 (0.24)
						111, 45	Number of monitoring visits at 1st year, Number of monitoring visits at 2nd year		5.65 (3.63), 4.13 (3.65)
					IVI	776, 60	Number of treatment visits at 1st year, Number of treatment visits at 2nd year		1.85 (1.26), 1.73 (0.92)
						603, 232	Number of monitoring visits at 1st year, Number of monitoring visits at 2nd year		5.11 (4.36), 3.78 (0.25)
					Laser photocoagulation	109, 6	Number of treatment visits at 1st year, Number of treatment visits at 2nd year		1.32 (0.72), 1.00 (0.00)
						54, 17	Number of monitoring visits at 1st year, Number of monitoring visits at 2nd year		4.35 (3.79), 3.94 (3.17)
Ruiz-Moreno 2016 [18]	mCNV or myopia without mCNV	Spain	EUR	2014	Group I: mCNV	137	Ophthalmologist visit	Mean (Q1–Q3); n (%)	7.60 (2.00–10.00); 95 (67.20)
							Retina specialists visit		10.70 (6.00–13.00); 125 (91.20)
							Optometrist visit		3.70 (1.00–4.00); 59 (43.10)
							Orthoptists visit		5.00 (5.00–5.00); 1 (0.70)

							Family doctor visit		7.00 (2.00–7.00); 24 (17.50)
							Primary nurse visit		3.00 (3.00–3.00); 1 (0.70)
							Occupational therapist visit		20.00 (20.00–20.00); 1 (0.70)
							Other specialists visit		14.20 (11.00–16.00); 10 (7.30)
							Emergency room visit		1.50 (1.00–1.00); 57 (41.70)
							Hospitalisation	n (%)	0 (0.00)
							Use of ambulances for hospitalisation		0 (0.00)
							Use of ambulances for emergencies		1 (1.80)
							Acquisition of assistive devices/aids		71 (51.80)
							Need for help with household chores		8 (5.80)
							Absenteeism (Days; Within the past 12 months)	Mean (Q1–Q3)	21.80 (0.00–8.50)
							Presenteeism (Days; Within the past 6 months)		6.70 (0.00–0.00)
					Group II: Myopia without mCNV	48	Ophthalmologist visit	Mean (Q1-Q3); n (%)	8.80 (3.00–7.00); 36 (75)
							Retina specialists visit		9.00 (3.00–8.00); 37 (77.10)
							Optometrist visit		7.20 (3.00–5.00); 25 (52.10)
							Orthoptists visit		NR; 0 (0.00)

							Family doctor visit		5.40 (3.00–10.00); 5 (10.40)
							Primary nurse visit		8.50 (5.00–8.50); 2 (4.20)
							Occupational therapist visit		NR; NR
							Other specialists visit		13.70 (9.00–19.00); 3 (6.30)
							Emergency room visit		1.70 (1.00–1.00); 12 (25)
							Hospitalisation	n (%)	2 (4.20)
							Use of ambulances for hospitalisation		0 (0.00)
							Use of ambulances for emergencies		0 (0.00)
							Acquisition of assistive devices/aids		21 (43.80)
							Need for help with household chores n (%)		1 (2.10)
							Absenteeism (Days; Within the past 12 months)	Mean (Q1–Q3)	0.00
							Presenteeism (Days; Within the past 6 months)		1.20 (0.00–0.00)
Zaour 2014 [19]	CNV secondary to PM, or mCNV	Canada	NR	NR	CNV secondary to PM, or mCNV	98	Retina specialist consultation, per year	Mean; n (%)	6; 88 (90)
							Emergency room visit	n (%)	11 (11.20)
							Hospitalisation		2 (2.00)

Abbreviations: AI – artificial intelligence, AMD - age-related macular degeneration, CEA - cost-effectiveness analysis, CI - confidence interval, CNV - choroidal neovascularisation, CNY - Chinese yuan, CUA - cost-utility analysis, FS-LASIK - femtosecond laser-assisted in situ keratomileusis, HCRU – healthcare resource utilisation, ICER - incremental cost-effectiveness ratio, ICUR - incremental cost-utility ratios, IVI - intravitreal antivascular endothelial growth factor injections, LASIK - (Laser-Assisted In Situ Keratomileusis), mCNV - myopic choroidal neovascularisation, MMD - myopic macular degeneration, NR - not reported, PM - pathologic myopia, PPPY - per patient per year, PRK - photorefractive keratectomy, Q1 - quarter 1, Q3 - quarter 3, QALYs - quality-adjusted life-years, RMB - renminbi, SD - standard deviation, SGD - Singapore dollar, SMILE - small incision lenticule extraction, TWD - Taiwanese dollar, USD - United States dollar, vPDT - verteporfin photodynamic therapy.
Note: None of the studies reported data on the life-years gained or incremental life-years.
^^ Conbercept demonstrated significant cost-effectiveness in pathological myopia treatment due to its dominance over ranibizumab, which was more expensive and less effective.
¶Administration (non-drug) cost per visit, including examination, diagnosis, and concomitant drug. ¶¶Cost per monitoring visit including diagnosis, examination, and others.
# Scenario 1: The number of injections in two arms were from the clinical trials; Scenario 2: The number of injections in two arms were from real-world studies. * per person. ## per 100,000 people screened.
Zheng 2013: Additional studies for different age groups have been taken from the consolidated table in Zhang et al. 2013. SP2: Singapore Prospective Study Programme; SEED: Singapore Epidemiology of Eye Disease Study.
Note: Values reported are in the currency reported in the original articles (unconverted and uninflated).

Supplementary Table S8. Health care costs and HCRU findings for all age groups

Health care costs									
Reference	Population	Country/Region	Currency	Cost year	Subgroups	Sample size	Description	Unit	Value
**Direct costs**									
Ma 2022 [21]	Myopia 5 to 50 years	Mainland China	CNY (1 USD = 6.6 CNY as of June 1st, 2016)	2016	Patients residing in middle social economic area (Anhui)				
					Treated with spectacles	1,415	Annual cost (discounted), per pair	Mean (SD)	238.20 (284.70)
						1,261	Annual cost, per patient		155.40 (210.30)
					Treated with OrthoK and RGPCL	11	Annual cost (discounted), per pair		4,673.40 (2,546.30)
						7	Annual cost for lenses		3,107.10 (1,632.10)
						9	Annual running cost		1,726.70 (1,264.60)
						5	Annual cost, per patient		3,762.00 (2,384.50)
					Treated with SCL	128	Annual cost, per patient		950.60 (1,166.70)
					Medical health service in hospital	577	Annual medical cost		109.50 (246.10)
					Medical health service in hospital	577	Traffic cost		37.10 (181.10)
					Myopic laser surgery	18	Medical cost		9,199.00 (6,392.60)
					Treatment outside hospital	616	Annual cost		238.20 (423.80)
					Preventing myopia	NR	Annual cost		424.60 (376.80)
					Patients residing in upper social economic area (Shanghai)				
					Treated with spectacles	2,135	Annual cost (discounted), per pair	Mean (SD)	555.40 (1,077.70)
						1,878	Annual cost, per patient		314.30 (706.60)
					Treated with OrthoK and RGPCL	57	Annual cost (discounted), per pair		5,902.40 (3,511.00)
						45	Annual cost for lenses		4,189.30 (4,042.30)
						56	Annual running cost		1,610.40 (1,093.20)
						40	Annual cost, per patient		5,822.10 (4,504.40)
					Treated with SCL	413	Annual cost, per patient		1,740.20 (2,355.50)
					Medical health service in hospital	553	Annual medical cost		154.80 (281.70)
					Medical health service in hospital	553	Traffic cost		22.10 (56.80)

					Myopic laser surgery	17	Medical cost		10,636.40 (6,225.30)
					Treatment outside hospital	334	Annual cost		356.10 (625.90)
					Preventing myopia	NR	Annual cost		568.20 (407.50)
					Patients residing in lower social economic area (Yunnan)				
					Treated with spectacles	1,627	Annual cost (discounted), per pair	Mean (SD)	470.90 (690.60)
						1,308	Annual cost, per patient		303.00 (550.80)
					Treated with OrthoK and RGPCL	38	Annual cost (discounted), per pair		6,465.60 (2,739.80)
						30	Annual cost for lenses		4,007.50 (2,344.70)
						47	Annual running cost		1,703.00 (1,194.20)
						27	Annual cost, per patient		5,669.70 (3,059.5)
					Treated with SCL	156	Annual cost, per patient		1,268.10 (1,462.60)
					Medical health service in hospital	673	Annual medical cost		170.20 (325.90)
					Medical health service in hospital	673	Traffic cost		59.50 (203.30)
					Myopic laser surgery	16	Medical cost		13,315.60 (6,734.90)
					Treatment outside hospital	527	Annual cost		385.30 (598.50)
					Preventing myopia	NR	Annual cost		386.90 (386.60)
Fricke 2022 [22]	Myopia all age groups	Australia & China	USD	2020	Australian myopia patients	NR	Lifetime cost of TMM	Mean (CI)	7,437.00 (4,953.00–10,740.00)
						NR	Lifetime cost of anti-myopia spectacles		7,280.00 (5,246.00–9,888.00)
						NR	Lifetime cost of low-dose atropine		NR
					Chinese myopia patients	NR	Lifetime cost of TMM		8,006.00 (3,026.00–13,707.00)
						NR	Lifetime cost of anti-myopia spectacles		NR
						NR	Lifetime cost of low-dose atropine		4,453.00 (2,136.00–9,115.00)
**Indirect costs**									

Ma 2022 [21]	Myopia 5 to 50 years	Mainland China	CNY (1 USD = 6.6 CNY as of June 1st, 2016)	2016	Mild to moderate visual impairment	24.8 million	Cost of loss of productivity, in billions	Mean (SD)	44.50 (NR)
					Severe visual impairment to blindness	4.1 million	Cost of loss of productivity, in billions		62.20 (NR)
					Patients residing in middle social economic area (Anhui)				
					Medical health service in hospital	541	Annual cost of working time loss	Mean (SD)	38.00 (138.20)
						505	Annual cost of accompanying		263.20 (1,026.90)
					Myopic laser surgery	18	Cost of working time loss		1,560.00 (1,962.80)
					Treatment outside hospital	664	Annual cost of working time loss		26.30 (57.70)
						628	Annual cost of accompanying		119.90 (367.30)
					Patients residing in upper social economic area (Shanghai)				
					Medical health service in hospital	526	Annual cost of working time loss	Mean (SD)	71 (308.80)
						498	Annual cost of accompanying		484.00 (3,245.50)
					Myopic laser surgery	17	Cost of working time loss		4,912.90 (5,565.50)
					Treatment outside hospital	373	Annual cost of working time loss		71.10 (165.90)
						365	Annual cost of accompanying		283.10 (1,386.80)
					Patients residing in lower social economic area (Yunnan)				
					Medical health service in hospital	638	Annual cost of working time loss	Mean (SD)	89.10 (430.40)
						590	Annual cost of accompanying		512.50 (1,486.50)
					Myopic laser surgery	16	Cost of working time loss		3,255.00 (3,973.60)
					Treatment outside hospital	575	Annual cost of working time loss		55.60 (173.20)
						552	Annual cost of accompanying		2,576.00 (54,608.90)
**HCRU**									
**Reference**	**Population**	**Country/Region**	**Currency**	**Cost year**	**Subgroups**	**Sample size**	**Description**	**Unit**	**Value**
Ma 2022 [21]	Myopia 5 to 50 years	Mainland China	CNY (1 USD = 6.6 CNY) as of June 1st, 2016	2016	Patients residing in middle social economic area (Anhui)				
					Overall	2,796	Hospital visit	%	20.60
						NR	Medical health service utilisation (aged 5 to 9)		64.20
							Medical health service utilisation (aged 10-14)		44.60
							Medical health service utilisation (aged 15 to 19)		23.80

							Medical health service utilisation (Other age intervals)	Range %	7.00–13.70
					Spectacles	1,968	Frequency of replacement, years	Mean (SD)	2.10 (1.60)
						NR	Medical health service utilisation (aged 5 to 9)	%	59.35
							Medical health service utilisation (aged 10-14)		82.12
							Medical health service utilisation (aged 15 to 19)		94.58
					OrthoK and RGPCL	13	Frequency of replacement, years	Mean (SD)	1.20 (0.80)
						NR	Medical health service utilisation (aged 5 to 9)	%	2.49
							Medical health service utilisation (aged 10-14)		3.11
							Medical health service utilisation (aged 15 to 19)		1.02
					SCL	NR	Medical health service utilisation (aged 5 to 9)	%	-
							Medical health service utilisation (aged 10-14)		0.62
							Medical health service utilisation (aged 15 to 19)		5.15
					Patients residing in upper social economic area (Shanghai)				
					Overall	3,175	Hospital visit	%	16.80
						NR	Medical health service utilisation (aged 5 to 9)		81.50
							Medical health service utilisation (aged 10-14)		55.40
							Medical health service utilisation (aged 15 to 19)		16.50
							Medical health service utilisation (Other age intervals)	Range %	6.00–9.80
					Spectacles	2,330	Frequency of replacement, years	Mean (SD)	2.40 (1.80)
						NR	Medical health service utilisation (aged 5 to 9)	%	62.34
							Medical health service utilisation (aged 10-14)		79.95

							Medical health service utilisation (aged 15 to 19)		92.96
					OrthoK and RGPCL	53	Frequency of replacement, years	Mean (SD)	1.80 (1.10)
						NR	Medical health service utilisation (aged 5 to 9)	%	3.89
							Medical health service utilisation (aged 10-14)		9.10
							Medical health service utilisation (aged 15 to 19)		3.79
					SCL	NR	Medical health service utilisation (aged 5 to 9)	%	-
							Medical health service utilisation (aged 10-14)		1.14
							Medical health service utilisation (aged 15 to 19)		8.64
					Patients residing in lower social economic area (Yunnan)				
					Overall	2,333	Hospital visit	%	28.80
						NR	Medical health service utilisation (aged 5 to 9)		73.30
							Medical health service utilisation (aged 10-14)		66.70
							Medical health service utilisation (aged 15 to 19)		37.40
							Medical health service utilisation (Other age intervals)	Range %	6.50–20.10
					Spectacles	1,645	Frequency of replacement, years	Mean (SD)	2.30 (1.80)
						NR	Medical health service utilisation (aged 5 to 9)	%	40.11
							Medical health service utilisation (aged 10-14)		81.31
							Medical health service utilisation (aged 15 to 19)		92.96
					OrthoK and RGPCL	39	Frequency of replacement, years	Mean (SD)	1.60 (0.90)
						NR	Medical health service utilisation (aged 5 to 9)	%	-
							Medical health service utilisation (aged 10-14)		5.50

							Medical health service utilisation (aged 15 to 19)		5.70
					SCL	NR	Medical health service utilisation (aged 5 to 9)	%	-
							Medical health service utilisation (aged 10-14)		1.24
							Medical health service utilisation (aged 15 to 19)		6.28

Abbreviations: CI - confidence interval, CNY - Chinese yuan, HCRU – healthcare resource utilisation, NR - not reported, OrthoK - orthokeratology, RGPCL - rigid gas-permeable contact lens, SCL - soft contact lens, SD - standard deviation, TMM - traditional myopia management, USD - United States dollar.

Note: Values reported are in the currency reported in the original articles (unconverted and uninflated).

Figures

Supplementary Figure S1. Categorisation of included studies based on age and outcomes

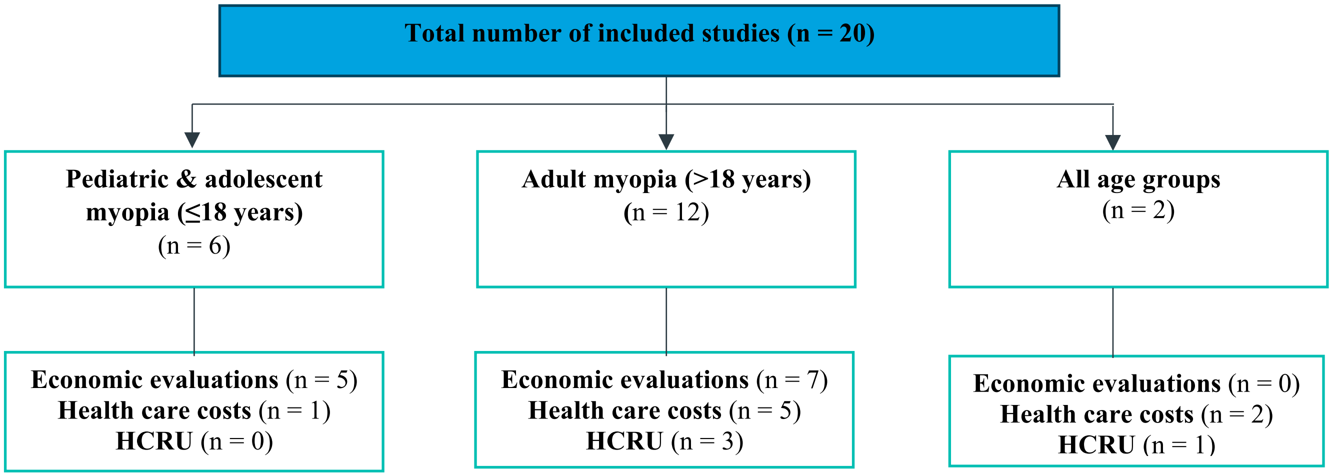

Abbreviation: HCRU - health care resource utilisation.

Supplementary Figure S2. Geographical distribution of identified SLR studies

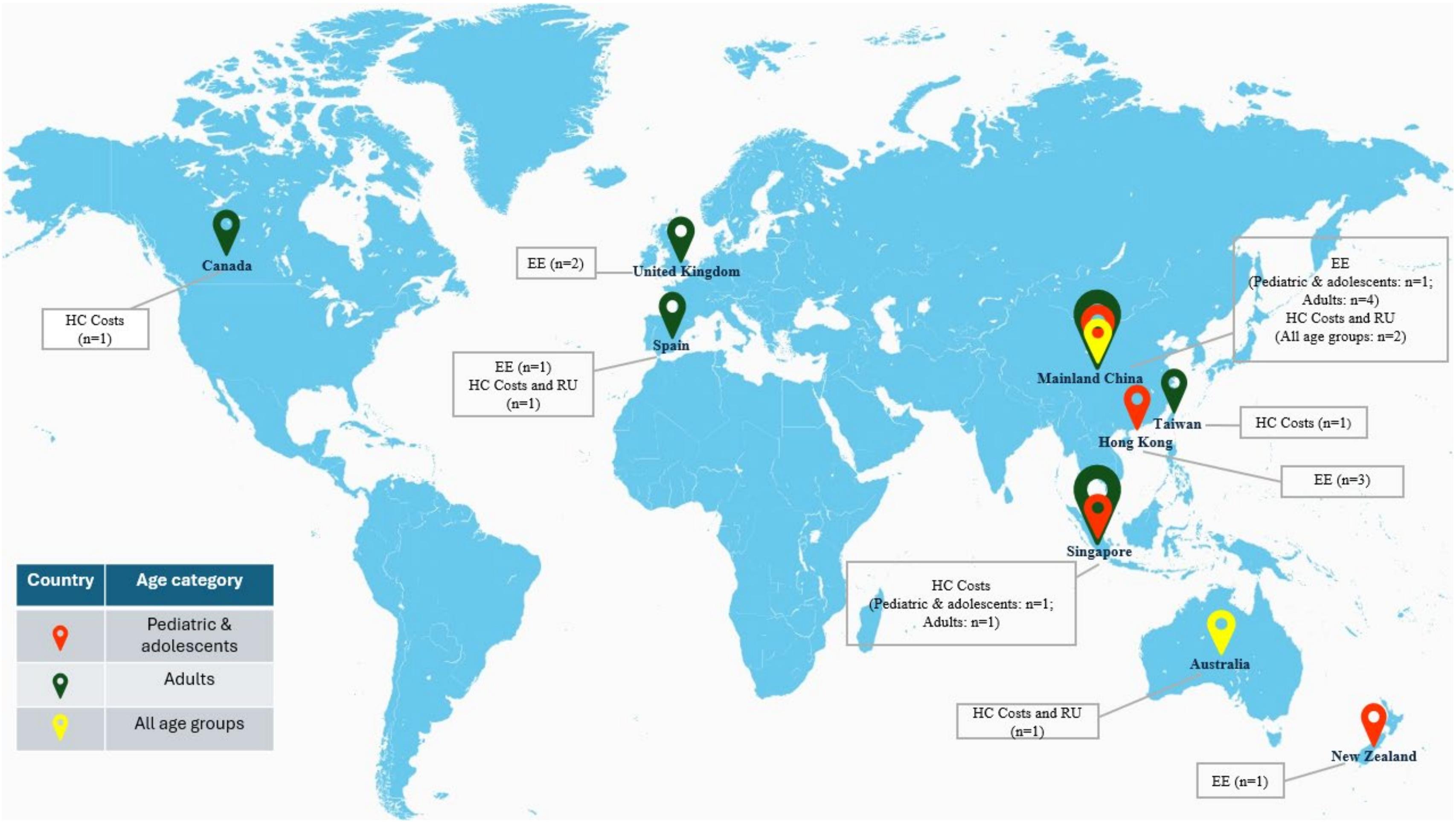

Abbreviations: EE - economic evaluations, HC costs - health care costs, RU - resource utilisation, SLR - systematic literature review.

Supplementary Figure S3. (a) Treatment and (b) monitoring visit costs in Taiwan

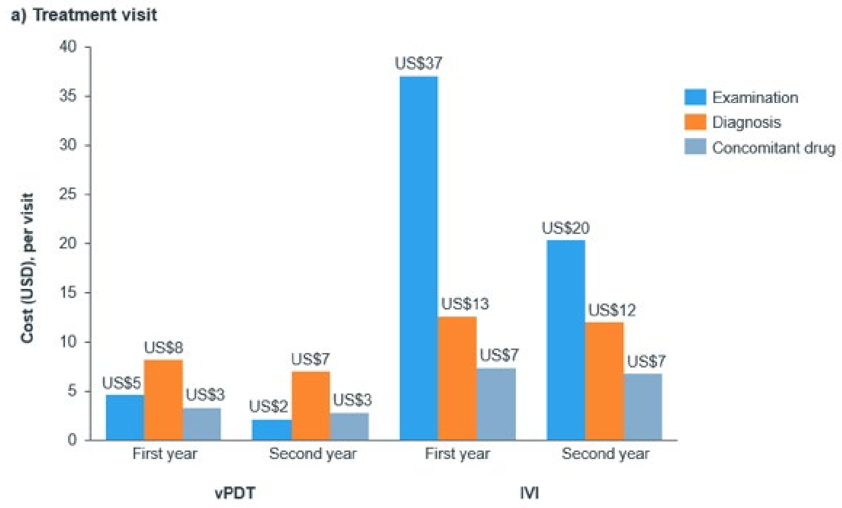

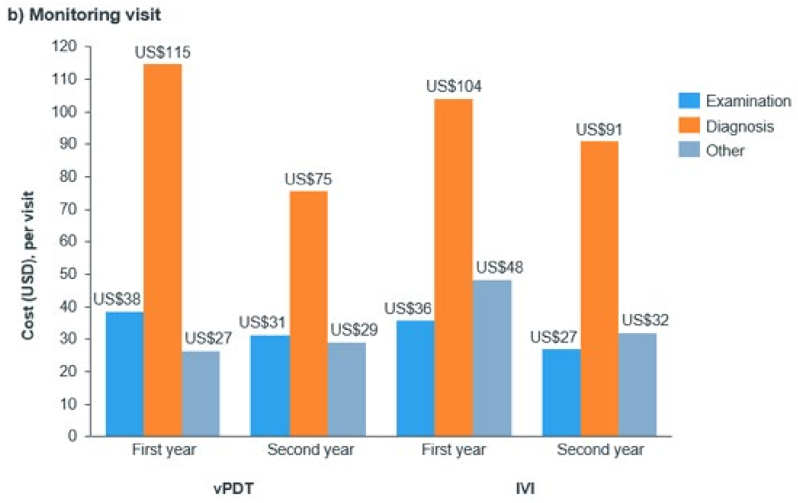

Figure a: Examination, diagnosis, and concomitant drug cost at treatment visits in Taiwan**; Figure b:** Examination, diagnosis, and other cost at monitoring visits in Taiwan.
Abbreviations: IVI - intravitreal antivascular endothelial growth factor injections, USD - United States dollar, vPDT - verteporfin photodynamic therapy**.**

Supplementary Figure S4. (a) Spectacles, (b) OrthoK/RGP, and (c) SCLs utilisation rate across different socioeconomic areas of mainland China

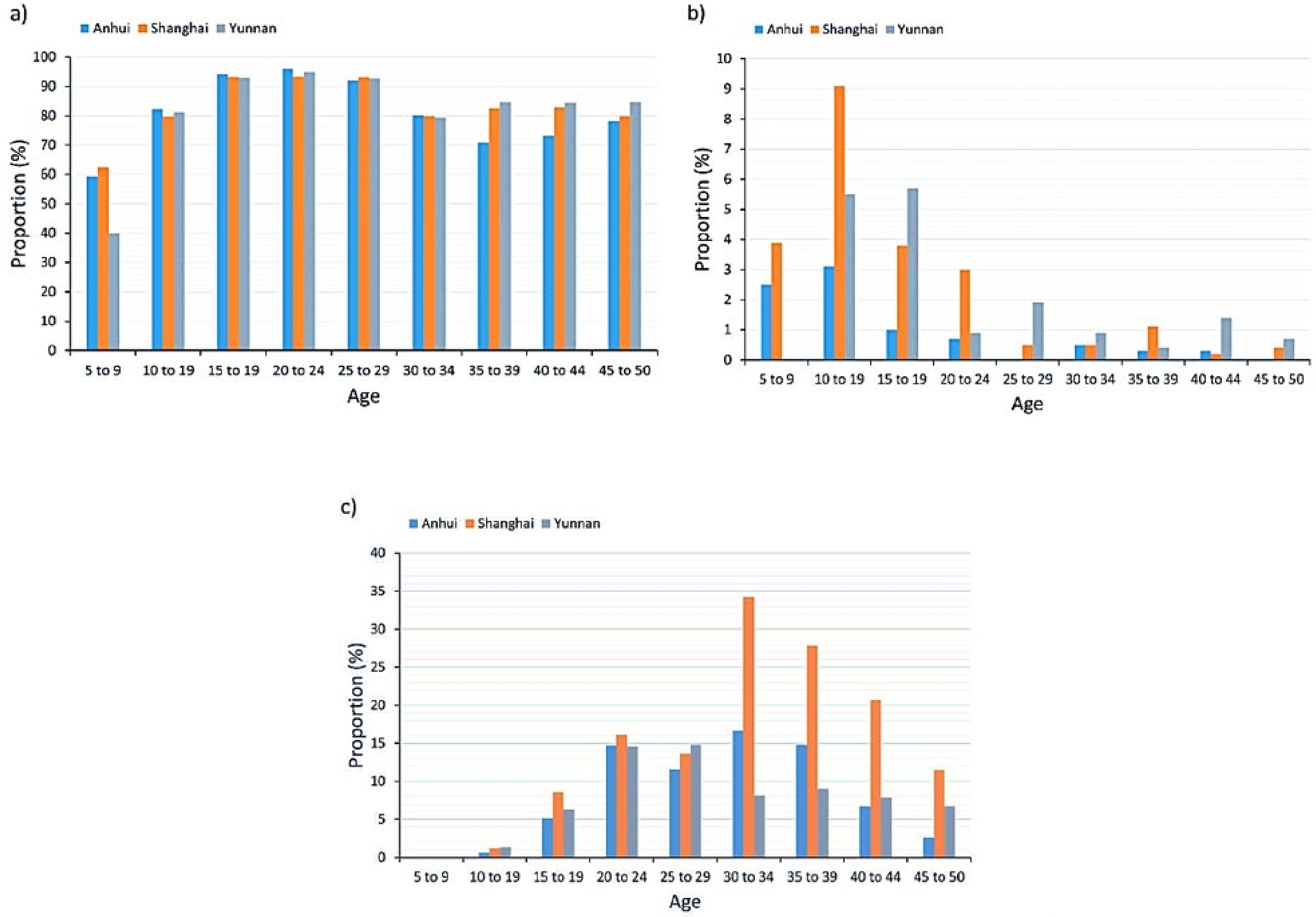

Figure a: Spectacles utilisation rate in three provinces; **Figure b:** Orthokeratology or RGP lenses utilisation rate in three provinces; **Figure c**: Soft contact lenses utilisation rate in three provinces.
Abbreviations: OrthoK - orthokeratology, RGP - rigid gas-permeable lenses, SCL - soft contact lenses.

PRISMA CHECKLIST

Section and Topic	Item #	Checklist item	Location where item is reported
**TITLE**			
Title	1	Identify the report as a systematic review.	
**ABSTRACT**			
Abstract	2	See the PRISMA 2020 for Abstracts checklist.	
**INTRODUCTION**			
Rationale	3	Describe the rationale for the review in the context of existing knowledge.	
Objectives	4	Provide an explicit statement of the objective(s) or question(s) the review addresses.	
**METHODS**			
Eligibility criteria	5	Specify the inclusion and exclusion criteria for the review and how studies were grouped for the syntheses.	
Information sources	6	Specify all databases, registers, websites, organisations, reference lists and other sources searched or consulted to identify studies. Specify the date when each source was last searched or consulted.	
Search strategy	7	Present the full search strategies for all databases, registers and websites, including any filters and limits used.	
Selection process	8	Specify the methods used to decide whether a study met the inclusion criteria of the review, including how many reviewers screened each record and each report retrieved, whether they worked independently, and if applicable, details of automation tools used in the process.	
Data collection process	9	Specify the methods used to collect data from reports, including how many reviewers collected data from each report, whether they worked independently, any processes for obtaining or confirming data from study investigators, and if applicable, details of automation tools used in the process.	
Data items	10a	List and define all outcomes for which data were sought. Specify whether all results that were compatible with each outcome domain in each study were sought (e.g. for all measures, time points, analyses), and if not, the methods used to decide which results to collect.	
	10b	List and define all other variables for which data were sought (e.g. participant and intervention characteristics, funding sources). Describe any assumptions made about any missing or unclear information.	
Study risk of bias assessment	11	Specify the methods used to assess risk of bias in the included studies, including details of the tool(s) used, how many reviewers assessed each study and whether they worked independently, and if applicable, details of automation tools used in the process.	
Effect measures	12	Specify for each outcome the effect measure(s) (e.g. risk ratio, mean difference) used in the synthesis or presentation of results.	
Synthesis methods	13a	Describe the processes used to decide which studies were eligible for each synthesis (e.g. tabulating the study intervention characteristics and comparing against the planned groups for each synthesis (item #5)).	
	13b	Describe any methods required to prepare the data for presentation or synthesis, such as handling of missing summary statistics, or data conversions.	
	13c	Describe any methods used to tabulate or visually display results of individual studies and syntheses.	
	13d	Describe any methods used to synthesize results and provide a rationale for the choice(s). If meta-analysis was performed, describe the model(s), method(s) to identify the presence and extent of statistical heterogeneity, and software package(s) used.	
	13e	Describe any methods used to explore possible causes of heterogeneity among study results (e.g. subgroup analysis, meta-regression).	
	13f	Describe any sensitivity analyses conducted to assess robustness of the synthesized results.	
Reporting bias assessment	14	Describe any methods used to assess risk of bias due to missing results in a synthesis (arising from reporting biases).	
Certainty assessment	15	Describe any methods used to assess certainty (or confidence) in the body of evidence for an outcome.	

Section and Topic	Item #	Checklist item	Location where item is reported
**RESULTS**			
Study selection	16a	Describe the results of the search and selection process, from the number of records identified in the search to the number of studies included in the review, ideally using a flow diagram.	
	16b	Cite studies that might appear to meet the inclusion criteria, but which were excluded, and explain why they were excluded.	
Study characteristics	17	Cite each included study and present its characteristics.	
Risk of bias in studies	18	Present assessments of risk of bias for each included study.	
Results of individual studies	19	For all outcomes, present, for each study: (a) summary statistics for each group (where appropriate) and (b) an effect estimate and its precision (e.g. confidence/credible interval), ideally using structured tables or plots.	
Results of syntheses	20a	For each synthesis, briefly summarise the characteristics and risk of bias among contributing studies.	
	20b	Present results of all statistical syntheses conducted. If meta-analysis was done, present for each the summary estimate and its precision (e.g. confidence/credible interval) and measures of statistical heterogeneity. If comparing groups, describe the direction of the effect.	
	20c	Present results of all investigations of possible causes of heterogeneity among study results.	
	20d	Present results of all sensitivity analyses conducted to assess the robustness of the synthesized results.	
Reporting biases	21	Present assessments of risk of bias due to missing results (arising from reporting biases) for each synthesis assessed.	
Certainty of evidence	22	Present assessments of certainty (or confidence) in the body of evidence for each outcome assessed.	
**DISCUSSION**			
Discussion	23a	Provide a general interpretation of the results in the context of other evidence.	
	23b	Discuss any limitations of the evidence included in the review.	
	23c	Discuss any limitations of the review processes used.	
	23d	Discuss implications of the results for practice, policy, and future research.	
**OTHER INFORMATION**			
Registration and protocol	24a	Provide registration information for the review, including register name and registration number, or state that the review was not registered.	
	24b	Indicate where the review protocol can be accessed, or state that a protocol was not prepared.	
	24c	Describe and explain any amendments to information provided at registration or in the protocol.	
Support	25	Describe sources of financial or non-financial support for the review, and the role of the funders or sponsors in the review.	
Competing interests	26	Declare any competing interests of review authors.	
Availability of data, code and other materials	27	Report which of the following are publicly available and where they can be found: template data collection forms; data extracted from included studies; data used for all analyses; analytic code; any other materials used in the review.	

REFERENCES

1. Inflation rate, average consumer prices

Annual percent change: International Monetary Fund; 2025. Available from: https://www.imf.org/external/datamapper/PCPIPCH@WEO/OEMDC/ADVEC/WEOWORLD.
2. Official exchange rate (LCU per US$, period average): World Bank Group; 2024. Available from: https://data.worldbank.org/indicator/PA.NUS.FCRF.
3. Li R, Zhang K, Li S-M, Zhang Y, Tian J, Lu Z, et al. Implementing a digital comprehensive myopia prevention and control strategy for children and adolescents in China: a cost-effectiveness analysis. The Lancet Regional Health–Western Pacific. 2023;38.
4. Agyekum S, Chan PP, Adjei PE, Zhang Y, Huo Z, Yip BH, et al. Cost-effectiveness analysis of myopia progression interventions in children. JAMA Network Open. 2023;6(11):e2340986-e.
5. Agyekum S, Zhang XJ, Chan PPM, Zhang Y, Huo Z, Yip BH, et al. Cost-Effectiveness Analysis of Atropine for Treating Myopia Progression in Children. Investigative Ophthalmology & Visual Science. 2023;64(8):821.
6. Lian J, McGhee S, Yap M, Sum R. Cost-effectiveness of myopia control by use of defocus incorporated multiple segments lenses: abridged secondary publication. Hong Kong Med J. 2023;29(6 Supplement 7).
7. Hong CY, Boyd M, Wilson G, Hong SC. Photorefraction screening plus atropine treatment for myopia is cost-effective: a proof-of-concept Markov analysis. Clinical Ophthalmology. 2022:1941-52.
8. Cui Z, Zhou W, Chang Q, Zhang T, Wang H, Meng X, et al. Cost-effectiveness of Conbercept vs. Ranibizumab for age-related macular degeneration, diabetic macular edema, and pathological myopia: population-based cohort study and Markov model. Frontiers in Medicine. 2021;8:750132.
9. LIU J, Xie S, Ni W, He X, Ren X, Wu J. PDG26 Cost-effectiveness of ranibizumab versus verteporfin photodynamic therapy in the treatment of choroidal neovasuclarization secondary to pathological myopia: from chinese societal perspective. Value in Health. 2019;22:S601.
10. Liu H, Li R, Zhang Y, Zhang K, Yusufu M, Liu Y, et al. Economic evaluation of combined population-based screening for multiple blindness-causing eye diseases in China: a cost-effectiveness analysis. The Lancet Global Health. 2023;11(3):e456-e65.
11. Zhang L, Lin Z, Zhang W, Xuan J. Cost-effectiveness analysis of conbercept versus ranibizumab for the treatment of myopic choroidal neovascularization (CNV) in China. ISPOR Europe. 2019.
12. Leteneux C, Claxton L, Malcolm W, Taylor M, Rathi H. Cost-Effectiveness of Ranibizumab for the Treatment of Visual Impairment Due to Choroidal Neovascularization Secondary to Pathologic Myopia in the United Kingdom. Value in Health. 2013;16(7):A505.
13. Balgos MJTD, Piñero DP, Canto-Cerdan M, Alió del Barrio JL, Alió JL. Comparison of the Cost-Effectiveness of SMILE, FS-LASIK, and PRK for Myopia in a Private Eye Center in Spain. Journal of Refractive Surgery. 2022;38(1):21-6.
14. Malcolm W, Leteneux C, Claxton L, Taylor M, Rathi H. The Budget Impact of Introducing Ranibizumab in England and Wales for the Treatment of Visual Impairment Due to Choroidal Neovascularization Secondary to Pathologic Myopia. Value in Health. 2013;16(7):A503.
15. Lim M, Gazzard G, Sim E, Tong L, Saw S. Direct costs of myopia in Singapore. Eye. 2009;23(5):1086-9.

16. Yang M-C, Chen Y-P, Tan EC-H, Leteneux C, Chang E, Chu CH, et al. Epidemiology, treatment pattern and health care utilization of myopic choroidal neovascularization: a population based study. Japanese journal of ophthalmology. 2017;61:159-68.
17. Zheng Y-F, Pan C-W, Chay J, Wong TY, Finkelstein E, Saw S-M. The economic cost of myopia in adults aged over 40 years in Singapore. Investigative ophthalmology & visual science. 2013;54(12):7532-7.
18. Ruiz-Moreno J, Roura M. Cost of myopic patients with and without myopic choroidal neovascularisation. Archivos de la Sociedad Española de Oftalmología (English Edition). 2016;91(6):265-72.
19. Zaour N, Heisel O, Leteneux C, Ma P. Canadian Burden Of Choroidal Neovascularization Secondary To Pathologic Myopia: Final Results. Value in Health. 2014;17(3):A284-A5.
20. Naidoo KS, Fricke TR, Sankaridurg P, Naduvilath T, Resnikoff S, Frick KD. Estimated global productivity loss from myopia. Investigative Ophthalmology & Visual Science. 2017;58(8):2404.
21. Ma Y, Wen Y, Zhong H, Lin S, Liang L, Yang Y, et al. Healthcare utilization and economic burden of myopia in urban China: a nationwide cost-of-illness study. Journal of global health. 2022;12.
22. Fricke. Factors affecting the lifetime cost of myopia and the impact of anti myopia treatments. 2022.
